# MFSD12 promotes proliferation, metastasis and invasion of hepatocellular carcinoma cells and its potential correlation with HAVCR2/LGALS9 immune checkpoint axis

**DOI:** 10.3389/fimmu.2025.1681887

**Published:** 2025-10-21

**Authors:** Kai Sun, Song Wen, Shou-jun Guo, Qing-hua Pan, Ke-run Wang

**Affiliations:** Department of Oncology, Ganzhou Cancer Hospital, The Affiliated Cancer Hospital of Gannan Medical University, Ganzhou, Jiangxi, China

**Keywords:** MFSD12, hepatocellular carcinoma, prognosis, immune infiltration, biomarkers

## Abstract

**Background:**

Major Facilitator Superfamily Domain-containing 12 (MFSD12) has emerged as a critical transmembrane protein with increasingly recognized roles in various cancers. The complex pathogenesis and therapeutic resistance of liver hepatocellular carcinoma (LIHC) present significant clinical challenges. This study investigates MFSD12’s potential involvement in LIHC progression.

**Methods and results:**

We performed an extensive pan-cancer analysis of MFSD12 utilizing integrated datasets from The Cancer Genome Atlas (TCGA), the Gene Expression Omnibus (GEO), and the ArrayExpress database. Our investigation focused on evaluating its prognostic significance, clinical implications, associated signaling pathways, immune cell infiltration, gene mutations, and sensitivity to chemotherapeutic agents. Through the application of R and various online analytical tools, our study demonstrated that MFSD12 expression levels were significantly higher in LIHC compared to other cancer types within the TCGA pan-cancer dataset. This finding highlights the specificity of MFSD12 expression in LIHC, a conclusion further validated by immunohistochemical analysis. Survival analysis indicated that this upregulation is associated with unfavorable clinical outcomes. Furthermore, single-cell RNA sequencing revealed that MFSD12 was predominantly expressed in tumor cells and innate lymphoid cells (ILCs) within the tumor microenvironment. Functional vitro studies showed MFSD12-siRNA treatment effectively suppressed LIHC cell proliferation, migration, and invasion. Mechanistically, MFSD12-siRNA enhanced E-cadherin while reducing vimentin, MMP-2, and MMP-9 levels. Further analyses revealed significant associations between MFSD12 expression and immune infiltration, immune checkpoint molecules, tumor mutation burden, and microsatellite instability in LIHC. Notably, MFSD12-siRNA decreased HAVCR2(TIM3) and its ligand galectin-9 (LGALS9) expression in LIHC cells.

**Conclusions:**

Our findings demonstrated that MFSD12 upregulation in LIHC strongly correlates with poor prognosis. This association was potentially attributed to MFSD12’s dual roles: promoting tumor cell proliferation, migration, and metastasis while critically modulating the tumor immune microenvironment, particularly through interaction with the HAVCR2/LGALS9 immune checkpoint axis.

## Introduction

Liver cancer treatment remains a significant challenge in medicine, particularly for hepatocellular carcinoma (LIHC/HCC), which is a leading cause of cancer-related deaths worldwide (1–3). While early-stage patients often have better outcomes, advanced LIHC still lacks effective treatment options due to its complex pathophysiological mechanisms and resistance to current therapies (4). Recent advances in understanding LIHC’s molecular mechanisms have led to promising developments in targeted treatments, such as those focusing on impaired signaling pathways like Notch, which has shown potential in preclinical studies (5–8). Additionally, microRNAs (miRNAs) play a key role in LIHC development, with research indicating their dysregulation affects critical cancer-related pathways, making miRNA-targeted therapies a potential future option for patients (9, 10).

The prognosis of LIHC is closely linked to its tumor immune microenvironment, as LIHC exhibits high heterogeneity where the immune microenvironment plays a pivotal role in tumor development, progression, and treatment response (11, 12). Studies have shown that the pattern of immune cell infiltration in the tumor immune microenvironment can significantly influence patient outcomes and treatment responses (13). In the immune microenvironment of LIHC, tumor-associated macrophages (TAMs) are one of the primary types of immune cells (1, 14). They promote tumor growth and immune escape by interacting with tumor cells and other immune cells (14). Additionally, the infiltration of immunosuppressive cells such as regulatory T cells (Tregs) and myeloid suppressor cells (MDSCs) is also associated with poor prognosis in LIHC (15). In recent years, single-cell sequencing technology has uncovered the heterogeneity of the immune microenvironment in LIHC, providing novel insights into understanding tumor immune escape mechanisms (16). Through single-cell analysis of LIHC samples, researchers have demonstrated that the infiltration patterns of different immune cell subpopulations are closely linked to patient survival rates and responses to immunotherapy (11). Furthermore, metabolic reprogramming in LIHC can affect its immune microenvironment, thereby influencing tumor progression and response to immunotherapy (4, 6). For example, dysregulated histidine metabolism can promote tumor growth and immune escape by altering the function of immune cells (11).

In terms of therapeutic strategies, combination therapy has demonstrated promising results. For instance, the combination of programmed cell death 1 (PD-1) inhibitors with chemotherapy drugs has shown both efficacy and safety in treating metastatic pancreatic ductal adenocarcinoma with liver metastases, offering new insights for LIHC treatment (13). Furthermore, nanomedicine has emerged as a potential therapeutic approach for LIHC (17). This field utilizes nanoscale or nanostructured materials for medical applications, and recent years have witnessed significant advancements in applying nanomedicine to LIHC treatment (17). Additionally, hepatocellular carcinoma stem cells (HSCs) represent an important therapeutic target (18). Given their close association with cancer metastasis, therapeutic strategies targeting HSCs may provide new hope for managing LIHC (18). Although LIHC treatment still faces numerous challenges, ongoing research into its molecular mechanisms and the continuous development of novel therapeutic approaches promise brighter prospects for future treatment outcomes (19–21).

MFSD12, or Major Facilitator Superfamily Domain-containing 12, is a transmembrane protein that plays a crucial role in cysteine import into melanosomes and lysosomes (22). This protein is essential for maintaining normal cystine levels, the oxidized dimer of cysteine, within these organelles (23). The function of MFSD12 is particularly important in pigmentation, as it contributes to pheomelanin synthesis, a type of melanin pigment, through cysteinyldopa production in melanosomes (24). The absence or downregulation of MFSD12 has been associated with darker pigmentation in both mice and humans, underscoring its regulatory role in skin color (22). Beyond pigmentation, MFSD12 has been implicated in lysosomal storage disorders, particularly cystinosis (25). Cystinosis is characterized by lysosomal cystine accumulation due to cystinosin dysfunction, the lysosomal cystine exporter. Research indicates that MFSD12 loss can decrease lysosomal cystine accumulation, a key pathological feature of cystinosis (25). However, despite this reduction, experimental models show no improvement in proximal tubular function, suggesting that while MFSD12 modulates cystine levels, it does not fully ameliorate the functional deficits associated with cystinosis (25).

MFSD12’s role in cancer has garnered significant attention in recent years. Studies have demonstrated that MFSD12 plays a crucial role in various cancers, particularly in the growth and progression of tumors including melanoma, breast cancer, and lung cancer (26). In melanoma, elevated MFSD12 expression correlates with enhanced cell proliferation and accelerated tumor growth by promoting cell cycle progression (23). Furthermore, MFSD12 upregulation activates the PI3K signaling pathway, and this proliferative effect can be reversed by PI3K inhibitors (23). In both breast and lung cancers, MFSD12 serves as a key oncogenic promoter, with its overexpression associated with poorer patient prognosis (26). As a cysteine transporter, MFSD12 contributes to lysosomal storage disease pathogenesis, suggesting its potential as a therapeutic target for inhibiting tumor progression, preventing metastasis, and improving treatment outcomes (26). Beyond its oncogenic functions, MFSD12 plays significant roles in lysosomal storage diseases. Research has established that MFSD12 is essential for cysteine import into melanosomes and lysosomes, with its deficiency leading to reduced cysteine levels in these organelles (22). In non-pigmented cells, MFSD12 deficiency similarly impairs lysosomal cysteine accumulation (22). Additionally, MFSD12 modulates glycolipid metabolism through lysosomal homeostasis regulation. In experimental cystinosis models, MFSD12 loss reduced cystine accumulation without restoring proximal tubule function (24). In liver cancer research, while MFSD12’s specific mechanisms remain understudied, its established roles in other cancers suggest potential functions (26). MFSD12 may promote tumor growth and metastasis by influencing metabolic and signaling pathways in cancer cells. As a cysteine transporter, it might also affect hepatocellular carcinoma progression by modulating oxidative stress responses in malignant cells. Given the established oncogenic roles of MFSD12 in various cancer types, we hypothesized that it might similarly contribute to the progression of LIHC. Nonetheless, comprehensive studies examining MFSD12 expression in relation to LIHC prognosis, immune cell infiltration, and its therapeutic implications remain lacking. This study aims to fill this gap by investigating the role of MFSD12 in LIHC progression and its interactions with the tumor immune microenvironment, ultimately identifying MFSD12 as a novel biomarker and potential therapeutic target.

This study systematically analyzed MFSD12 expression and its association with LIHC prognosis using multidimensional data from the TCGA, GEO, and ICGC databases. Immunohistochemical validation confirmed significantly increased MFSD12 expression in LIHC specimens. Mutational, single-cell, and pharmacological analyses of MFSD12 in LIHC have yielded insights into the role of MFSD12 within this context. *In vitro* functional studies assessed the effects of MFSD12 on LIHC cell proliferation, invasion, metastasis, as well as E-cadherin, vimentin, MMP-2, and MMP-9 expression levels. Furthermore, we investigated MFSD12’s influence on the tumor immune microenvironment in LIHC. Functional analyses validated the relationship between MFSD12 expression and both HAVCR2 and its ligand galectin-9 (LGALS9). Our findings provided novel insights into MFSD12’s critical role in LIHC pathogenesis.

## Materials and methods

### Data collection and preprocessing

The comprehensive pan-cancer genomic datasets were retrieved from The Cancer Genome Atlas (TCGA, https://portal.gdc.cancer.gov/) databases. For comparative analysis, normal tissue expression profiles were obtained from the Genotype-Tissue Expression project (GTEx, http://gtexportal.org/) (27). Among them, 110 normal liver samples from GTEx, 50 paired normal tissues adjacent to HCC (PNTAH) samples, 371 HCC samples, and the corresponding clinical data of TCGA liver cancer (TCGA-LIHC) were chosen for the subsequent analysis. To enhance the robustness of our findings, we supplemented our dataset with multiple LIHC cohorts from the Gene Expression Omnibus (GEO, https://www.ncbi.nlm.nih.gov/geo/), including: GSE144269 (70 hepatocellular carcinoma tissues and 70 adjacent normal liver tissues), GSE76427 (115 hepatocellular carcinoma tissues and 52 normal liver tissues), GSE104580 (147 hepatocellular carcinoma tissues), GSE116174 (64 hepatocellular carcinoma tissues), GSE14520 (22 hepatocellular carcinoma tissues and 22 paired non-tumor tissues, 42 hepatocellular carcinoma tissues and 22 paired non-tumor tissues), GSE54236 (81 hepatocellular carcinoma tissues and 80 non-tumor liver tissues) and GSE109211 (140 hepatocellular carcinoma tissues) (28). Additionally, the E_TABM_36 (57 hepatocellular carcinoma tissues, 3 hepatocellular adenomas tissues and 5 non-tumoral tissues) was acquired from the ArrayExpress database. The MFSD12 immunohistochemical (IHC) staining data were downloaded from the Human Protein Atlas (HPA, http://www.proteinatlas.org), which provides proteomics-based IHC results for multiple proteins across diverse tissues (29, 30). All expression data were processed as transcripts per million (TPM) and normalized through log2(TPM + 1) transformation. Data imputation was performed using the missForest R package to address missing values (31). We implemented rigorous quality control measures, identifying outliers via the interquartile range (IQR) method, where values beyond Q1 - 1.5×IQR or Q3 + 1.5×IQR were winsorized to the nearest valid observation (32). Final sample selection required both complete RNA-seq profiles and associated clinical annotations. To ensure comparability across the multiple datasets integrated from TCGA, GEO, and ArrayExpress, we implemented a rigorous data harmonization pipeline. All gene expression data were uniformly processed as transcripts per million (TPM) and log2(TPM + 1) transformed. While a formal batch effect correction (e.g., ComBat) was not applied due to the inherent heterogeneity of public datasets and the focus on within-dataset analyses for key validations (e.g., TCGA for discovery, independent GEO cohorts for validation), we employed stringent quality control measures. These included the removal of outliers and the requirement for complete clinical annotation, which collectively minimize the impact of technical variability on our core findings. The consistent results observed across independent cohorts further support the robustness of our conclusions against potential batch effects.

### Prognosis analysis

The survival analysis was performed using Kaplan-Meier methodology to evaluate overall survival (OS), progression-free survival (PFS), Disease-free survival (DFS) and disease-specific survival (DSS) stratified by MFSD12 expression levels. This analysis was implemented through the R statistical packages “DESeq2” and “limma” (33, 34). For prognostic assessment, the “timeROC” package was employed to compute 1-year, 3-year, and 5-year survival probabilities, with corresponding receiver operating characteristic (ROC) curves generated and their respective area under the curve (AUC) values quantified (35). Furthermore, survival patterns associated with varying MFSD12 expression levels in LIHC were validated using datasets from the GEO database (28, 36). Both univariate and multivariate Cox proportional hazards regression models were subsequently applied to identify significant prognostic factors. To improve the robustness of prognostic evaluation, we further performed bootstrap resampling validation across multiple cohorts (TCGA, GSE116174, GSE144269, GSE14520, and GSE76427). The stability of hazard ratios was assessed by repeating the Cox regression analysis in 1,000 bootstrap samples for each dataset.

### Genomic alterations and mutation profiles

The transcriptomic profiles (STAR-counts), somatic mutation data (MAF format), and associated clinical metadata for pan-cancer types were obtained from TCGA database (https://portal.gdc.cancer.gov). To ensure data completeness, we implemented stringent inclusion criteria, selecting only those specimens with concurrent availability of RNA-seq expression profiles, mutation data, and clinical annotations. This rigorous filtering process yielded a final cohort that were deemed suitable for downstream analytical procedures. The somatic mutation profiles of LIHC patients were analyzed and graphically represented using the maftools package within the R statistical environment (37).

### DNA methylation analysis

The EWAS Data Hub (https://ngdc.cncb.ac.cn/ewas/datahub/index) served as a comprehensive repository for DNA methylation analysis, encompassing 115,852 samples across 528 distinct diseases (38). In addition, the Shiny Methylation Analysis Resource Tool (SMART) App (http://www.bioinfo-zs.com/smartapp/) is a tool that integrates Infinium Human Methylation 450K BeadChip data, RNA sequencing data, and clinical information for 33 cancer types derived from TCGA dataset (39). We utilized the two public databases to investigate MFSD12 methylation patterns in LIHC patients. The 35 CpG sites selected for analysis were located within the promoter region (TSS1500, TSS200, 5’UTR, 1st Exon) of the MFSD12 gene, as methylation in these regions is known to exert the most pronounced effects on transcriptional regulation. Our analysis focused on examining the correlation between MFSD12 methylation status and its transcriptional expression and clinical characteristics, along with assessing its prognostic significance for survival status in affected individuals.

### Enrichment analysis

To elucidate the functional role of MFSD12 in LIHC, comprehensive gene ontology (GO) and Kyoto Encyclopedia of Genes and Genomes (KEGG) pathway analyses were conducted (40). The GO analysis, an established computational approach in functional genomics, systematically characterized LIHC-associated biological processes, molecular functions, and cellular components (41). Gene Set Enrichment Analysis (GSEA) was employed to explore the underlying molecular mechanisms, with this method being particularly valuable for identifying statistically significant differences in predefined gene sets across distinct biological conditions (42). All computational analyses were implemented using specialized bioinformatics tools: the ClusterProfiler package in R facilitated the GO and KEGG analyses, while GSEA version 4.1.0 was utilized for the enrichment analysis.

### Immune correlation analysis

To analyze the correlation between MFSD12 and immune cell infiltration, stromal, immune, and estimate scores, as well as Tumor Mutational Burden (TMB) and Microsatellite Instability (MSI) in LIHC from TCGA, GEO and ArrayExpress database, the R packages “GSVA”, “immunedeconv”, “estimate”, “ggplot2”, “pheatmap”, and “ggstatsplot” were used. The analysis involved eight of the latest algorithms, including ssGSEA, xCell, CIBERSORT, EPIC, TIMER, MCP-counter, TIMER and quanTIseq. Additionally, we examined the relationship between MFSD12 and 150 marker genes identified for five immune pathways (chemokine (41), receptor (18), MHC (21), immunoinhibitor (24), and immunostimulator (46)) (43–45). The statistical analysis information was visualized by R version 4.3.0.

### Single−cell expression analysis

The single-cell RNA sequencing data in.h5 format, along with the corresponding annotation files, were obtained from the TISCH database (46). Subsequent bioinformatics analyses were performed using the MAESTRO and Seurat R packages for data processing and quality control. Dimensionality reduction and cell clustering were accomplished through the application of the t-SNE algorithm. In the analysis of the GSE140228 dataset, transcriptomic profiles from 41 LIHC specimens were systematically processed, including normalization, feature selection, and unsupervised clustering to identify distinct cellular subpopulations. The analytical pipeline incorporated rigorous quality assessment metrics to ensure data reliability throughout the computational workflow. The sample metadata for the single-cell RNA sequencing dataset (GSE140228) is provided in **Supplementary Table S1**. While detailed clinical annotations such as TNM stage and etiology were not available for this public dataset, the table summarizes critical information including the patient source, tissue origin (e.g., tumor core, adjacent liver, blood), and cell type (CD45+ immune cells). This information is essential for understanding the cellular heterogeneity and tissue context of the samples analyzed.

### Drug sensitivity of MFSD12 in LIHC

The drug sensitivity data were obtained from well-established and publicly available databases, including the Cancer Therapeutics Response Portal (CTRP v2.0) (https://portals.broadinstitute.org/ctrp.v2.1/), the PRISM database (https://www.theprismlab.org/), and the Genomics of Drug Sensitivity in Cancer (GDSC) database (https://www.ancerrxgene.org/). Spearman’s rank correlation analysis was performed to evaluate associations between gene expression and the sensitivity of 217 pharmacological compounds, including kinase inhibitors, epigenetic regulators, and chemotherapeutic agents. All computational analyses were conducted in R version 4.3.0 using the tidyverse package for data manipulation, pRRophetic for drug response prediction, and ComplexHeatmap for generating visual representations (47).

### Tissue samples and immunohistochemistry

Nineteen pairs of LIHC and adjacent liver tissues were collected from Ganzhou Cancer Hospital, with the study protocol reviewed and approved by the Ethics Committee (Reference No. 2025Kelunshen121). Patient diagnoses were confirmed through histopathological analysis. Detailed clinical data are presented in **Supplementary Table S2**. Inclusion criteria comprised histologically confirmed LIHC and complete clinical data, while exclusion criteria included ambiguous pathological diagnoses, incomplete clinical characteristics, and prior treatment with more than three lines of drug therapy. Initially, the tissue samples were fixed in 10% formalin, embedded in paraffin, sectioned to a thickness exceeding four millimeters, dewaxed, hydrated, and subjected to antigen retrieval (1:100; Boster, China). Subsequently, sections were treated with a secondary antibody conjugated to horseradish peroxidase (ZSGB-Bio, China), followed by staining with 3,3′-diaminobenzidine (DAB) and hematoxylin. The integrated optical density (IOD) for each section was quantified using Image-Pro Plus 6.0 software (Media Cybernetics, USA), based on images captured from each slice.

### Cell culture

Human hepatocellular carcinoma HEP3B2.1–7 cell line were purchased from Sangon Biotech (Shanghai, China). HEP 3B2.1–7 cells were cultured in grown in MEM (Procell, PM150410) supplemented with 10% fetal bovine serum (FBS, Gibco, USA), with additional 1% penicillin/streptomycin (Solarbio, China) at 37˚C humidified incubator containing 5% CO^2^.

### siRNA transfection

The small interfering RNA (siRNA) sense used were as follows:

siMFSD12-Homo-578: sense:5’-GCCCGUUCAUCGUGAUCUUTT-3’, antisense:5’- AAGAUCACGAUGAACGGGCTT-3’.
siMFSD12Homo-1407: sense:5’-GAGCUUCUUGGAUAAGGUGTT-3’, antisense:5’- UCAUUUGGAUACAGGACCCTT-3’.
siMFSD12-Homo-1198: sense:5’-GGGAGGAACAUGACCUACUTT-3’, antisense:5’- AGUAGGUCAUGUUCCUCCCTT -3’.
siMFSD12-Homo-1021: sense:5’-GUGGGCAUACUGUACAUGATT-3’, antisense:5’- UCAUGUACAGUAUGCCCACTT-3’.

The sense of negative control RNA (NC) was as follows:

siNC: sense:5’-UUCUCCGAACGUGUCACGUTT-3’, antisense:5’- ACGUGACACGUUCGGAGAATT -3’.

HEP 3B2.1–7 cells were seeded in 6-well plates 1 day prior to transfection and cultured until reaching 80-90% confluence. Cells were transfected with siRNA or NC using Lipofectamine transfection reagent (KeyGEN, China) following the manufacturer’s protocol. Total RNA and protein were extracted 24 hours post-transfection for subsequent analyses. Subsequently, we established six experimental groups for the forthcoming study: CTRL (HEP 3B2.1–7 cells without additional treatment), siNC, si-MFSD12-1 (siMFSD12-Homo-578), si-MFSD12-2 (siMFSD12-Homo-1407), si-MFSD12-3 (siMFSD12-Homo-1198), and si-MFSD12-1 (siMFSD12-Homo-1021). The siRNA sequence identified as the most effective, si-MFSD12-3, was subsequently chosen for further functional assays.

### Real−time fluorescence quantitative PCR

Total RNA was extracted utilizing the RNA Isolater Total RNA Extraction Reagent (VAZYME), following the manufacturer’s instructions. Subsequently, RNA was reverse transcribed into complementary DNA (cDNA) using the HiScript^®^ II Q RT SuperMix for qPCR (+gDNA wiper) (VAZYME). Each cDNA sample was then subjected to analysis using the ChamQ SYBR qPCR Master Mix (VAZYME). The relative gene expression levels were determined using the 2^-△△CT method. The primer sequences used were as follows:

MFSD12: forward, 5’-CACCCAAGACATCAGCATC-3’; reverse, 5’-TGGAATAGCAGTGAGAACA-3’, 111bp.
GAPDH: forward, 5’-ATGGGGAAGGTGAAGGTCGGAGT-3’; reverse, 5’- TAGTTGAGGTCAATGAAGGGGTC-3’.

### Western blot analysis

Following transfection, the cells underwent two washes with PBS and were subsequently lysed on ice using RIPA lysis buffer. Protein concentrations were quantified employing the BCA protein assay kit (GBCBIO, China). The samples were then subjected to separation via 10% SDS-PAGE and transferred onto a nitrocellulose membrane (Biofroxx, Germany). Membranes were blocked with 5% skim milk for two hours and incubated overnight at 4 °C with primary antibodies. The membranes were washed three times with TBST for 10 minutes each. Subsequently, they were incubated with an HRP-conjugated goat anti-mouse IgG antibody (1:10000, Boster, China). The membranes were washed again in the same manner and developed. The primary antibodies utilized in the Western blot analysis included: anti-rabbit MFSD12 (1:1000, Boster, China), anti-mouse GAPDH (1:30000, Proteintech, China), anti-rabbit E-cadherin (1:40000, Proteintech, China), anti-mouse Vimentin (1:40000, Proteintech, China), anti-rabbit MMP2 (1:1000, BIOSS, China), anti-rabbit MMP9 (1:1000, Affinity, China), anti-rabbit LGALS9 (1:1000, Abmat, China), and anti-rabbit HAVCR2 (1:1000, Boster, China).

### Cell proliferation assay

Cell proliferation was assessed utilizing the CCK-8 assay kit (HYCEZMBIO, China). Post-transfection, cells were plated in 96-well plates at a density of 3000 cells per well. Subsequently, 10 μL of CCK-8 solution was introduced to each well at various time intervals (24 hours, 36 hours, and 48 hours), followed by incubation at 37 °C for 1 hour. The optical density (OD) of each well was determined at a wavelength of 450 nm using a microplate reader (Thermo Scientific, USA).

### Transwell migration and invasion assay

A 24-well Transwell chamber equipped with 8 µm pore size membranes (Corning, USA) was prepared with 100 μL of Matrigel (Corning, USA). A serum-free medium containing transfected cells (HEP 3B2.1-7, 6×10^4^ cells per well) was added to the upper inserts, while the lower chambers were filled with 600 μL of medium supplemented with 20% FBS. Following a 24-hour incubation period, the lower chambers were fixed with 4% paraformaldehyde for 60 minutes and subsequently stained with 0.5% crystal violet for 20 minutes. Images from five high-power fields per membrane were captured to quantify the number of migrating or invading cells.

### Statistical analysis methods

The statistical analyses were conducted using R software (version 4.3.0), incorporating multiple analytical approaches to evaluate the data. Fold-change (FC) and hazard ratio (HR) metrics were calculated, along with P-values derived from Log-rank tests. Correlation assessments were performed using both Spearman and Pearson methods, while group comparisons were analyzed through Wilcoxon tests, t-tests (for two-group comparisons), and one-way ANOVA (for multiple-group comparisons). Survival outcomes were assessed via Kaplan-Meier curves and log-rank tests, with statistical significance defined as *p* < 0.05. In graphical representations, significance levels were denoted by asterisks: * (*P* < 0.05), ** (*P* < 0.01), *** (*P* < 0.001), and **** (*P* < 0.0001).

## Results

### Evaluation of MFSD12 mRNA expression and its association with clinical parameters in LIHC

**Figure 1:**
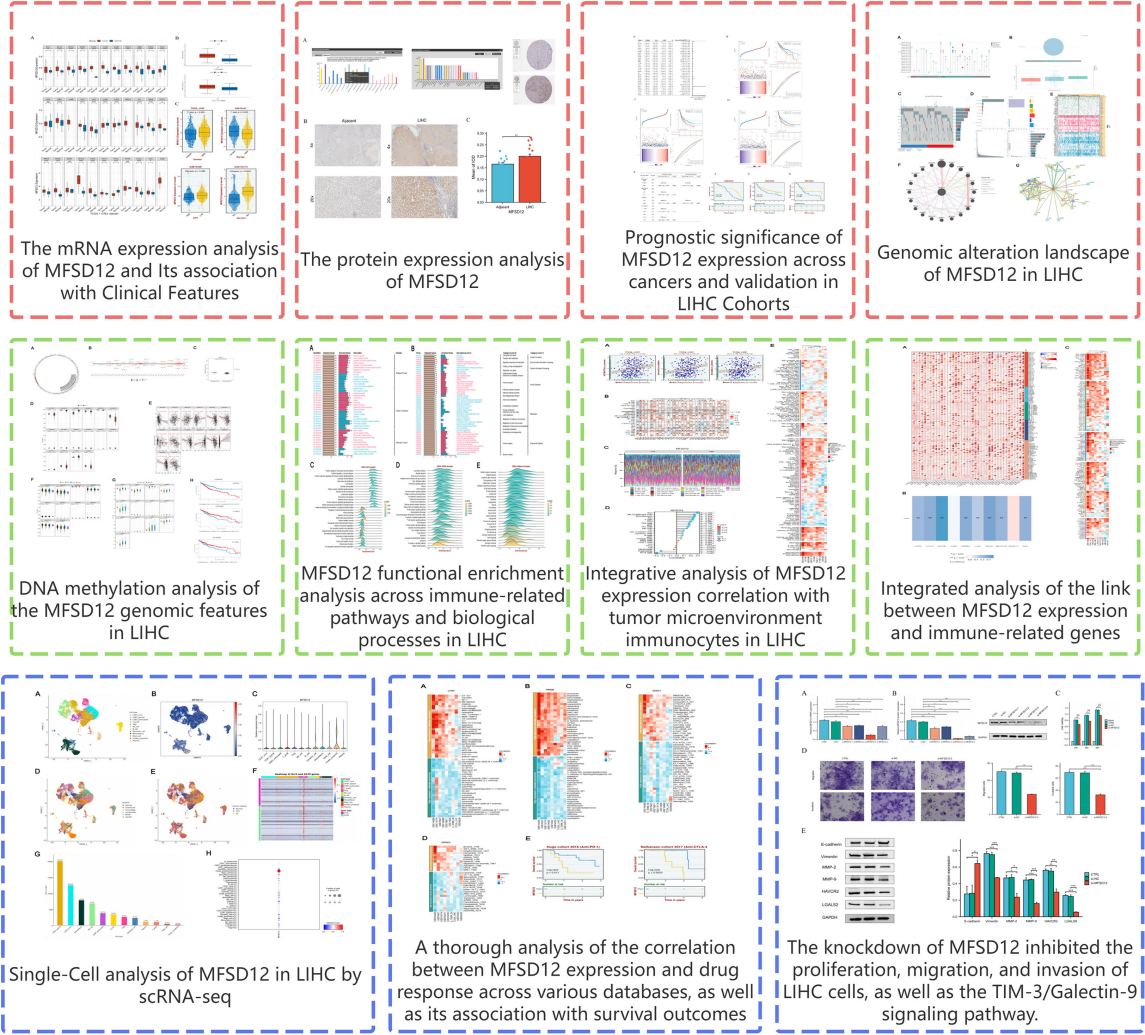
The research flowchart of this study.

**Figure 2:**
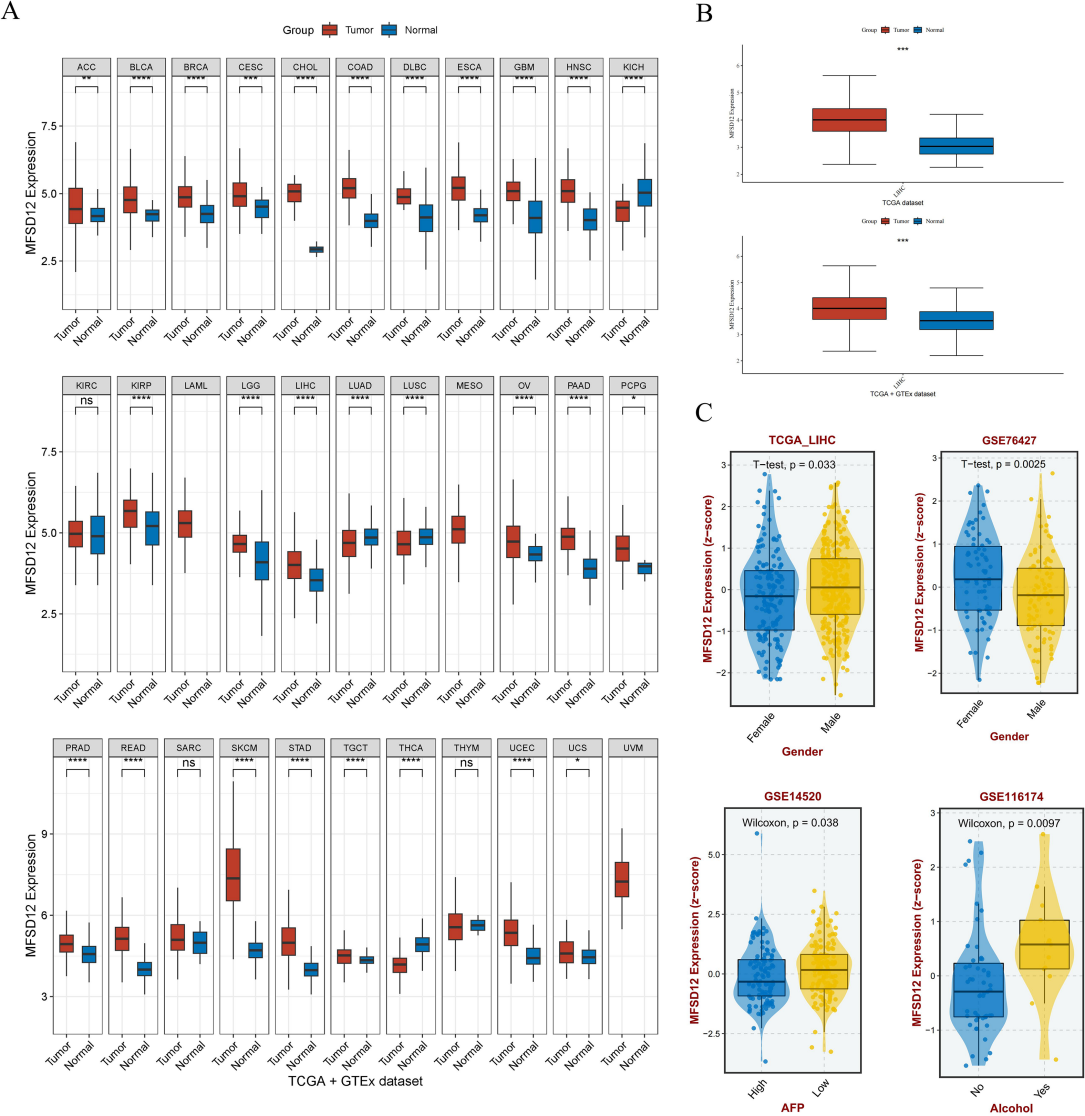
The mRNA expression analysis of MFSD12 and Its association with Clinical Features. **(A)** Differential expression analysis of MFSD12 between pan-cancer tissues and adjacent normal tissues in TCGA and GETx database. **(B)** Differential expression analysis of MFSD12 between tumor tissues and normal tissues in LIHC based on TCGA and GETx database. **(C)** Association of MFSD12 expression with clinical parameters in LIHC. *P < 0.05, **P < 0.01, ***P < 0.001, ****P < 0.0001. MFSD12, Major Facilitator Superfamily Domain-containing 12; LIHC, liver hepatocellular carcinoma; TCGA, The Cancer Genome Atlas; GEO, Gene Expression Omnibus, AFP, Alpha-fetoprotein. "ns" stands for "not significant".

The study design flowchart is shown in **Figure 1**. To comprehensively characterize the pan-cancer mRNA expression profile of MFSD12, transcriptomic data from 33 cancer types were sourced from TCGA database, while corresponding normal tissue data were obtained from the GTEx database. A comparative analysis of MFSD12 differential expression between malignant and normal tissues was conducted across all cancer types. The analysis demonstrated that MFSD12 mRNA expression was significantly elevated in the majority of cancer tissues relative to normal tissues (**Figure 2A**). This elevated expression pattern was notably observed in adrenocortical carcinoma (ACC), bladder urothelial carcinoma (BLCA), breast invasive carcinoma (BRCA), cervical squamous cell carcinoma and endocervical adenocarcinoma (CESC), cholangiocarcinoma (CHOL), colon adenocarcinoma (COAD), lymphoid neoplasm diffuse large B-cell lymphoma (DLBC), esophageal carcinoma (ESCA), glioblastoma (GBM), head and neck squamous cell carcinoma (HNSC), kidney papillary cell carcinoma (KIRP), stomach adenocarcinoma (STAD), kidney renal clear cell carcinoma (KIRC), lower grade glioma (LGG), liver hepatocellular carcinoma (LIHC), ovarian serous cystadenocarcinoma (OV), pancreatic adenocarcinoma (PAAD), pheochromocytoma and paraganglioma (PCPG), prostate adenocarcinoma (PRAD), rectum adenocarcinoma (READ), and skin cutaneous melanoma (SKCM), testicular germ cell tumors (TGCT), and uterine corpus endometrial carcinoma (UCEC), as well as uterine carcinosarcoma (UCS). Conversely, in kidney chromophobe (KICH), lung adenocarcinoma (LUAD), lung squamous cell carcinoma (LUSC), and thyroid carcinoma (THCA), MFSD12 expression was lower in cancer tissues than in the corresponding normal tissues. Subsequently, we examined the expression of MFSD12 in LIHC and its associations with various clinical parameters. Analysis of tumor samples from the TCGA-LIHC cohort revealed significantly elevated MFSD12 expression compared to adjacent normal tissues (*P* < 0.001) (**Figure 2B**). This pattern of overexpression was further validated in the combined TCGA and GTEx dataset, where it remained statistically significant (*P* < 0.001). Clinical correlation analyses demonstrated notable associations between MFSD12 expression and LIHC clinical parameters (**Figure 2C**). Gender-specific stratification indicated a male-predominant overexpression in the TCGA-LIHC cohort (*P* = 0.033) and a female-predominant overexpression in the GSE76427 dataset (*P* = 0.0025). Furthermore, higher MFSD12 expression was associated with lower alpha-fetoprotein (AFP) levels in the GSE14520 cohort (*P* = 0.038) and was correlated with a history of alcohol consumption in the GSE116174 cohort (*P* = 0.0097).

In addition, analysis of GSE196434 revealed that MFSD12 expression showed differential patterns before and after treatment, with variations observed across gender groups (**Supplementary Figure S1**). These results may partly explain the discrepancies in gender-associated expression trends observed between TCGA-LIHC and GSE76427 cohorts, possibly due to differences in baseline clinical characteristics and treatment status.

### MFSD12 protein expression analysis in public database and vitro experimental validation

**Figure 3:**
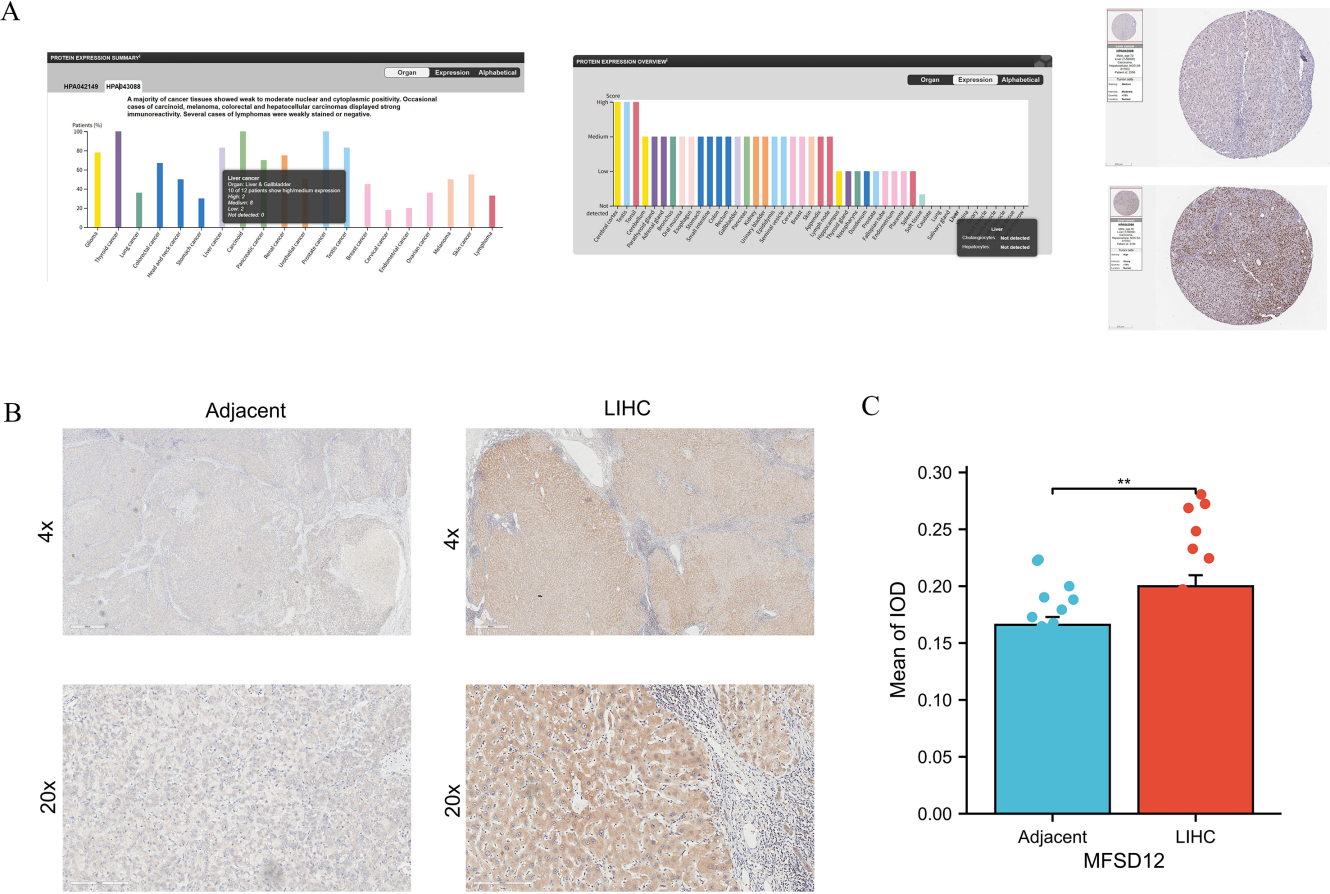
The protein expression analysis of MFSD12. **(A)** Pan-cancer protein expression profile of MFSD12 and representative IHC staining of tissue microarrays in HPA database. **(B)** IHC analysis of MFSD12 in LIHC tumor tissues and paired adjacent non-tumor liver tissues. **(C)** Quantification of immunostains for MFSD12 by IOD analysis. *P < 0.05, **P < 0.01. IHC, immunohistochemistry; HPA, Human Protein Atlas; LIHC, liver hepatocellular carcinoma; IOD, integrated optical density;.

To corroborate the aforementioned findings, an analysis was conducted utilizing the HPA database. The investigation revealed that the majority of cancerous tissues exhibited weak to moderate positivity in both nuclear and cytoplasmic compartments (**Figure 3A**). Notably, specific instances of carcinoid tumors, melanomas, COAD, and LIHC exhibited pronounced immunoreactivity, as evidenced by the strong and moderate positivity observed in two LIHC patients, as illustrated in **Figure 3A**. In contrast, normal liver tissues displayed no expression of the MFSD12 protein. Additionally, IHC analysis was performed to assess MFSD12 expression in 19 pairs of LIHC tumor tissues and their corresponding adjacent normal tissues. The IHC staining analysis indicated that MFSD12 proteins were predominantly localized within the cytoplasm of LIHC cells, with brown staining denoting positive expression (**Figure 3B**). In normal tissues, MFSD12 proteins were either weakly expressed or not expressed. Subsequent to immunohistochemical analysis, it was determined that MFSD12 protein levels, as quantified by the integrated optical density (IOD) value, were significantly elevated in LIHC tissues compared to adjacent non-tumor tissues (*P* < 0.05) (**Figure 3C**). To further validate the differential expression of MFSD12, we analyzed the GSE213797 dataset. Consistent with our previous findings, the expression level of MFSD12 was significantly altered following a specific intervention (Post) compared to the baseline state (Pre) (**Supplementary Figure S2**), reinforcing the dynamic regulation of MFSD12 in LIHC-related conditions. To statistically evaluate the consistency between our IHC results and the HPA database, two pathologists independently scored the samples in a blinded manner. A Kappa consistency test showed substantial agreement between the two evaluations (Kappa value = 0.75, p < 0.001), validating the reliability of our protein expression findings.

### Prognostic significance of MFSD12 expression in pan-cancer and LIHC

**Figure 4:**
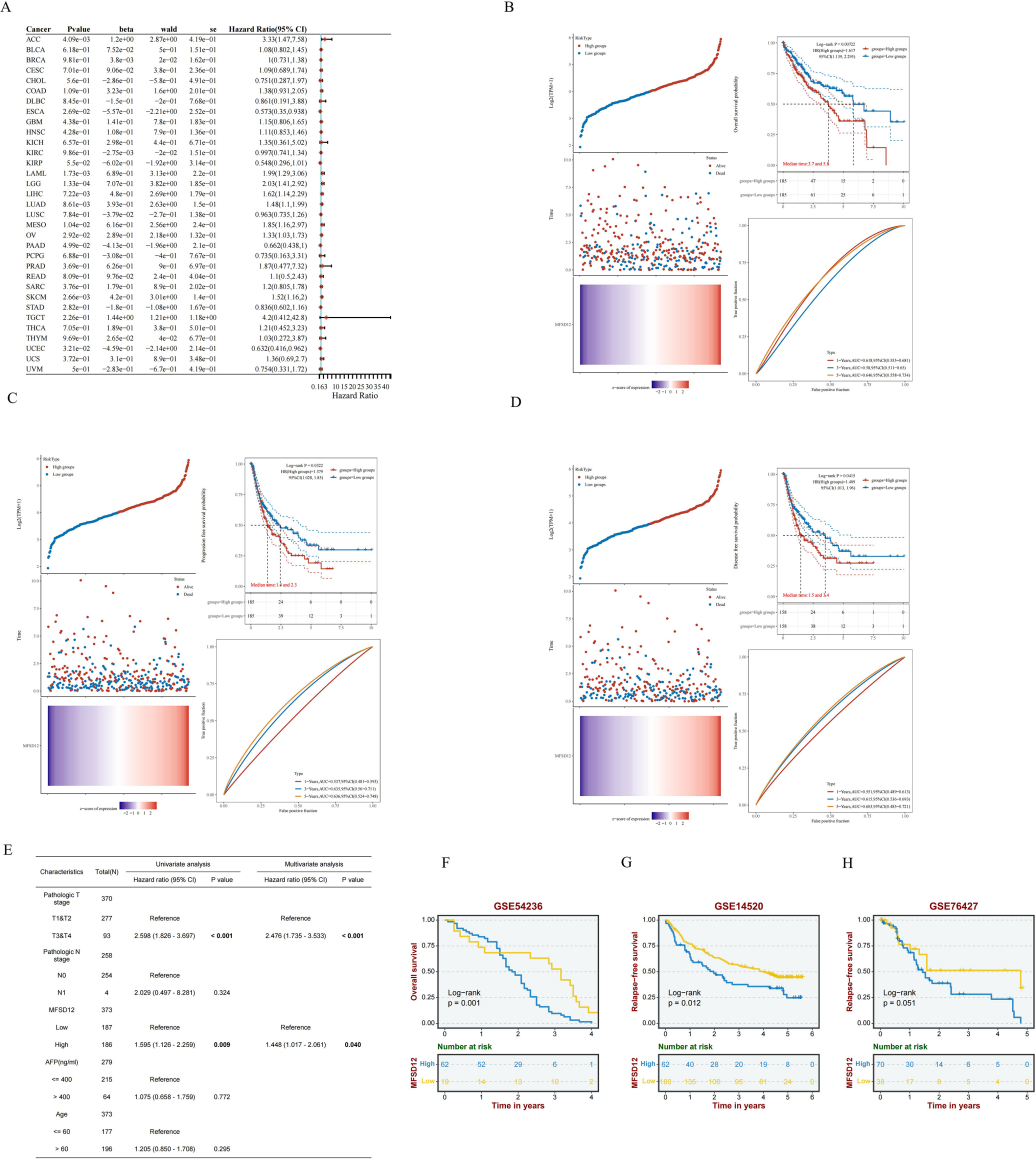
Prognostic significance of MFSD12 expression across cancers and validation in LIHC Cohorts. **(A)** A pan-cancer Cox regression analysis was performed to assess MFSD12 expression. **(B)** OS analysis of MFSD12 in TCGA-LIHC data. **(C)** PFS analysis of MFSD12 in TCGA-LIHC data. **(D)** DFS analysis of MFSD12 in TCGA-LIHC data. **(E)** The prognostic significance of MFSD12 expression in LIHC patients was evaluated through both univariate and multivariate analyses. **(F–H)** Independent validation using external GEO cohorts corroborated the prognostic significance of MFSD12 in LIHC. AUC, Area Under Curve; CI, Confidence Interval; DFS, Disease-Free Survival; GEO, Gene Expression Omnibus; HR, Hazard Ratio; LIHC, Liver Hepatocellular Carcinoma; OS, Overall Survival; PFS, Progression-Free Survival; RFS, Relapse-Free Survival; ROC, Receiver Operating Characteristic; TCGA, The Cancer Genome Atlas; TPM, Transcripts Per Million.

Now that we have identified the aberrant expression of MFSD12 in LIHC, we next analyzed the relationship between MFSD12 expression and the prognosis of LIHC. We first performed univariate Cox regression analysis across 33 cancer types, which indicated that high MFSD12 expression was significantly associated with poorer overall survival (OS) in ACC (HR = 3.33, *p* = 4.09e−03), LAML (HR = 1.99, *p* = 1.73e−03), LGG (HR = 2.03, *p* = 1.33e−04), LIHC (HR = 1.62, *p* = 7.22e−03), LUAD (HR = 1.48, *p* = 8.61e−03), MESO (HR = 1.85, *p* = 1.04e−02), and OV (HR = 1.33, *p* = 2.92e−02), whereas it inversely correlated with favorable OS in ESCA (HR = 0.573, *p* = 2.69e−02), KIRP (HR = 0.548, p=5.50e−02), and UCBC (HR = 0.632, p=3.21e−02)(**Figure 4A**). In LIHC (**Figures 4B–D**), high MFSD12 expression predicted worse clinical outcomes: OS (HR = 1.62, *p* = 0.007), Progression-free survival (PFS) (HR = 1.38, *p* = 0.032), Disease-free survival (DFS)(HR = 1.41, *p* = 0.041). Univariate Cox regression identified high MFSD12 expression as a significant risk factor for OS (HR = 1.585, *p* = 0.009), which remained significant in multivariate analysis adjusting for pathologic T stage, AFP level, and age (HR = 1.448, *p* = 0.040), with pathologic T stage (T3/T4 *vs*. T1/T2) serving as an independent prognostic factor (*p* < 0.001) (**Figure 4E**). This multivariate analysis adjusted for key prognostic factors available in the dataset. We acknowledge that other important clinical variables such as liver cirrhosis status and etiology could further refine the prognostic model; however, consistent data for these parameters were not available for the entire cohort. The time-dependent receiver operating characteristic (ROC) analysis was performed to evaluate the predictive accuracy of MFSD12 for patient survival at 1, 3, and 5 years. In the TCGA-LIHC cohort, the area under the curve (AUC) values were 0.61, 0.58, and 0.65 for 1, 3, and 5 years, respectively. Similar analyses in the GEO cohorts (GSE116174, GSE144269, GSE14520, GSE76427) yielded varying AUC values across different time points (**Supplementary Figure S3**), providing a comprehensive assessment of the predictive power of MFSD12. Validation analyses using three independent GEO datasets (GSE54236, GSE14520, GSE76427) consistently showed that patients with high MFSD12 expression had shorter median OS than low-expression groups (*p* < 0.05) (**Figures 4F–H**). Bootstrap validation consistently confirmed the prognostic significance of MFSD12 across independent LIHC cohorts, with hazard ratios remaining stable across resampled datasets (**Supplementary Figure S4**). We further validated the prognostic value of MFSD12 across multiple independent GEO cohorts. Kaplan-Meier survival analysis demonstrated that high MFSD12 expression was consistently associated with poorer overall survival in datasets GSE116174 (*P* = 0.43), GSE144269 (*P* = 0.19), GSE14520 (*P* = 0.087), and GSE76427 (*P* = 0.099) (**Supplementary Figure S5**). Although the statistical significance varied among cohorts, the trend towards worse survival in the high-expression group was evident, underscoring the potential of MFSD12 as a robust prognostic marker in LIHC.

### Genomic alterations and protein interaction networks of MFSD12 in LIHC

**Figure 5:**
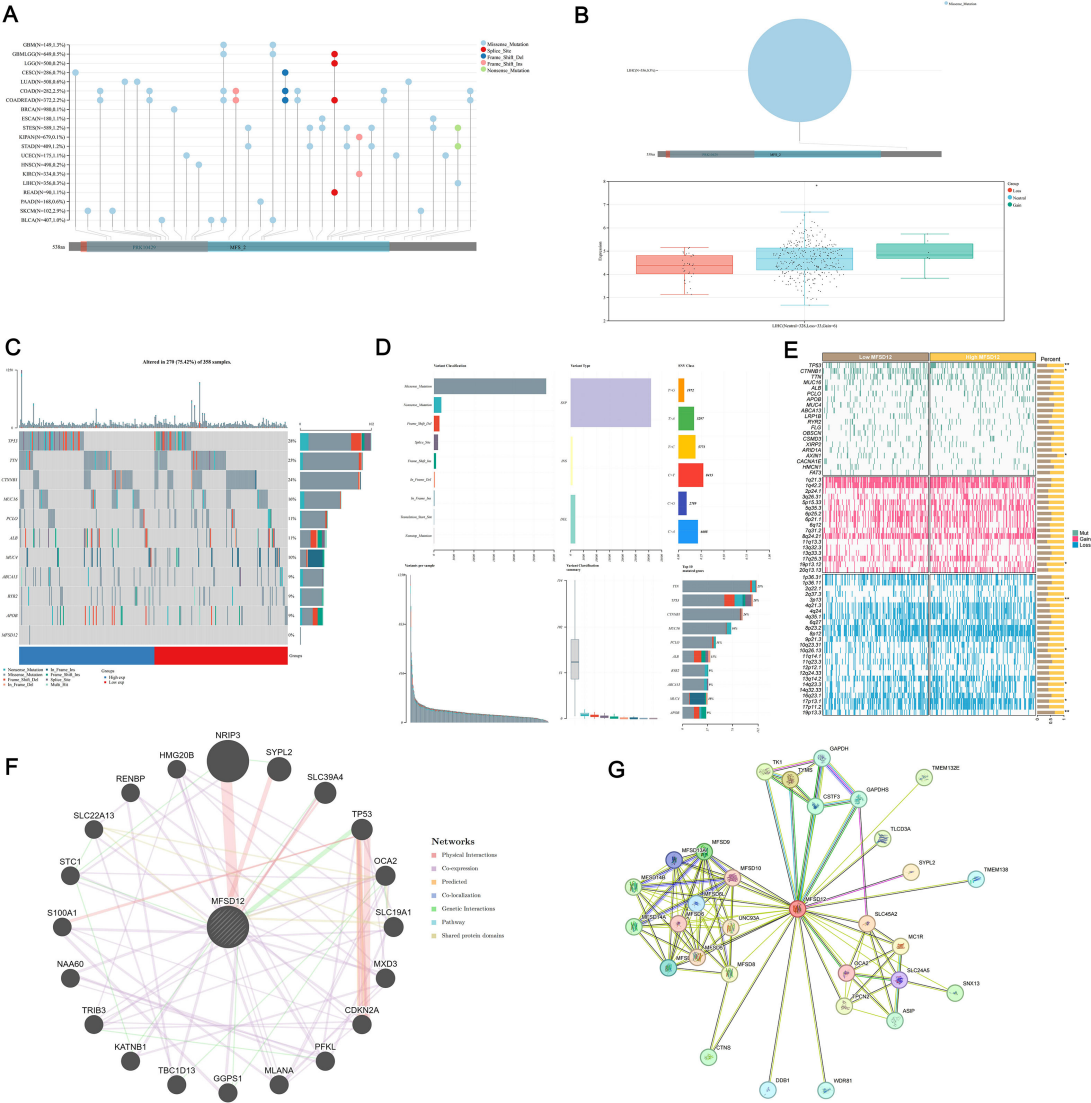
Genomic alteration landscape of MFSD12 in LIHC. **(A)** Mutation spectrum of MFSD12 in pan-cancer analysis. **(B)** CNV analysis of MFSD12 in LIHC. **(C)** Genomic Landscape of mutations in MFSD12 within LIHC. **(D)** Classification profile of MFSD12 genetic variants in LIHC. **(E)** Comparative mutation profiling in MFSD12 low- and high-expressing subpopulations. **(F)** Protein-protein interaction network analysis of MFSD12 in LIHC. **(G)** Gene Co- Expression Network Correlated with MFSD12 Expression Patterns in LIHC. *P < 0.05, **P < 0.01. CC, Cholangiocarcinoma; CNV, Copy Number Variation; LIHC, Liver Hepatocellular Carcinoma; MFSD12, Major Facilitator Superfamily Domain Containing 12; SNV, Single Nucleotide Variant; TCGA, The Cancer Genome Atlas; WES, Whole Exome Sequencing.

To further investigate the role of MFSD12 expression in LIHC, we analyzed the genetic mutation status of MFSD12 in LIHC as well as its associations with other proteins. Comprehensive genomic profiling uncovered low somatic mutation rates of MFSD12 across over 20 cancer types, with missense mutations emerging as the predominant variant type (e.g., LIHC: 0.3%, GBM: 1.3%, SKCM: 2.9%, COAD: 2.5%) (**Figure 5A**). Structural characterization using lollipop plots revealed that MFSD12 mutations were primarily localized to the PRK10429 domain and MFS_2 transporter motif. Notably, in LIHC, the somatic missense mutation rate was 0.3%, with no nonsense or frameshift mutations identified (**Figure 5B**). Given the exceptionally low somatic mutation rate of MFSD12 (0.3%) in the LIHC cohort, a meaningful statistical analysis stratifying mutation frequency by clinical stage (e.g., Stage I-IV) and assessing its association with disease progression using Fisher’s exact test was not feasible. The limited number of mutation events precludes a robust stratification analysis, indicating that the prognostic role of MFSD12 in LIHC is driven primarily by its expression levels rather than by genetic alterations. Copy number variation (CNV) analysis in LIHC demonstrated a predominance of neutral CNVs (328/367 samples), with infrequent copy number losses (33 samples) and gains (6 samples). These CNVs exhibited a weak correlation with mRNA expression levels (**Figure 5B**). Oncoplot analysis further confirmed that MFSD12 mutations were infrequent and did not co-cluster with high- or low-expression states, suggesting that its prognostic role in LIHC is likely driven by expression rather than coding sequence alterations (**Figure 5C**). Among these genetic alterations, missense mutations represented the predominant variant classification. At the nucleotide level, single nucleotide polymorphisms (SNPs) were the most prevalent mutation type, with T>G substitutions emerging as the most frequent among all single nucleotide variants (SNVs) (**Figure 5D**). The top 10 mutated genes displayed distinct mutation frequencies: TTN (28%), TP53 (25%), CTNNB1 (24%), MUC16 (16%), PCLO (11%), ALB (11%), RYR2 (9%), ABCA13 (9%), MUC4 (10%), and APOB (9%) (**Figure 5D**). In LIHC, comparative analysis between high- and low-MFSD12 expression groups revealed distinct somatic mutation landscapes. The top mutated genes in both groups included TP53, TTN, and CTNNB1, whereas MFSD12 mutation rates remained low and showed no significant difference between expression groups (**Figures 5C, E**). Genes such as TP53, CTNNB1, and AXIN1 exhibited significant differences in mutation frequencies between high- and low-MFSD12 expression groups. Additionally, genomic regions including 19p13.12 (gains) and 3p13, 10q26.13, 14q23.3, 17p13.1, and 19p13.3 (losses) displayed significant disparities in copy number gains/losses between high- and low-MFSD12 expression tumors (**Figure 5E**). Using the STRING and GeneMANIA platforms, we constructed comprehensive functional interaction networks to elucidate the molecular landscape of MFSD12. STRING analysis revealed a 32-node protein-protein interaction (PPI) network primarily composed of phylogenetically conserved transporters and metabolic enzymes, including multiple members of the Major Facilitator Superfamily (MFSD9, MFSD10, MFSD13A, MFSD14A, MFSD14B, MFSD5, MFSD6, MFSD8, MFSD11), glycolytic regulators (GAPDH, GAPDHS, TK1, TYMS), and transmembrane ion channels (SLC45A2, TPCN2) (**Figure 5F**). Notably, physical interactors also included pigmentation-related proteins (MC1R, OCA2, SLC24A5), implicating potential roles in cellular homeostasis and nutrient transport. GeneMANIA analysis further partitioned these interactions into distinct functional modules: physical associations (SYPL2, TMEM138, SNX13), co-expression networks (HMG20B, STC1, TP53), and genetic interactions (epistasis; S100A1, SLC19A1, NRIP3) (**Figure 5G**). Pathway enrichment analysis demonstrated significant overrepresentation in biological processes including glycolytic flux (GAPDH, PFKL), redox regulation (SLC45A2, MC1R), and cell cycle checkpoint control (CDKN2A, TRIB3). Structural domain characterization identified conserved transmembrane motifs shared between MFSD12 and its interacting partners (UNC93A, ASIP, CTNS), suggesting evolutionary conservation of its transport function. Collectively, these networks position MFSD12 as a hub node integrating metabolic signaling with stress response pathways, orchestrating crosstalk among transporters, epigenetic regulators, and oncogenic effectors. The modular architecture of these interactions provides mechanistic insights into MFSD12-mediated tumor progression, likely through coordinated regulation of nutrient homeostasis and cellular stress resilience.

### DNA methylation analysis of MFSD12 in LIHC patients

**Figure 6:**
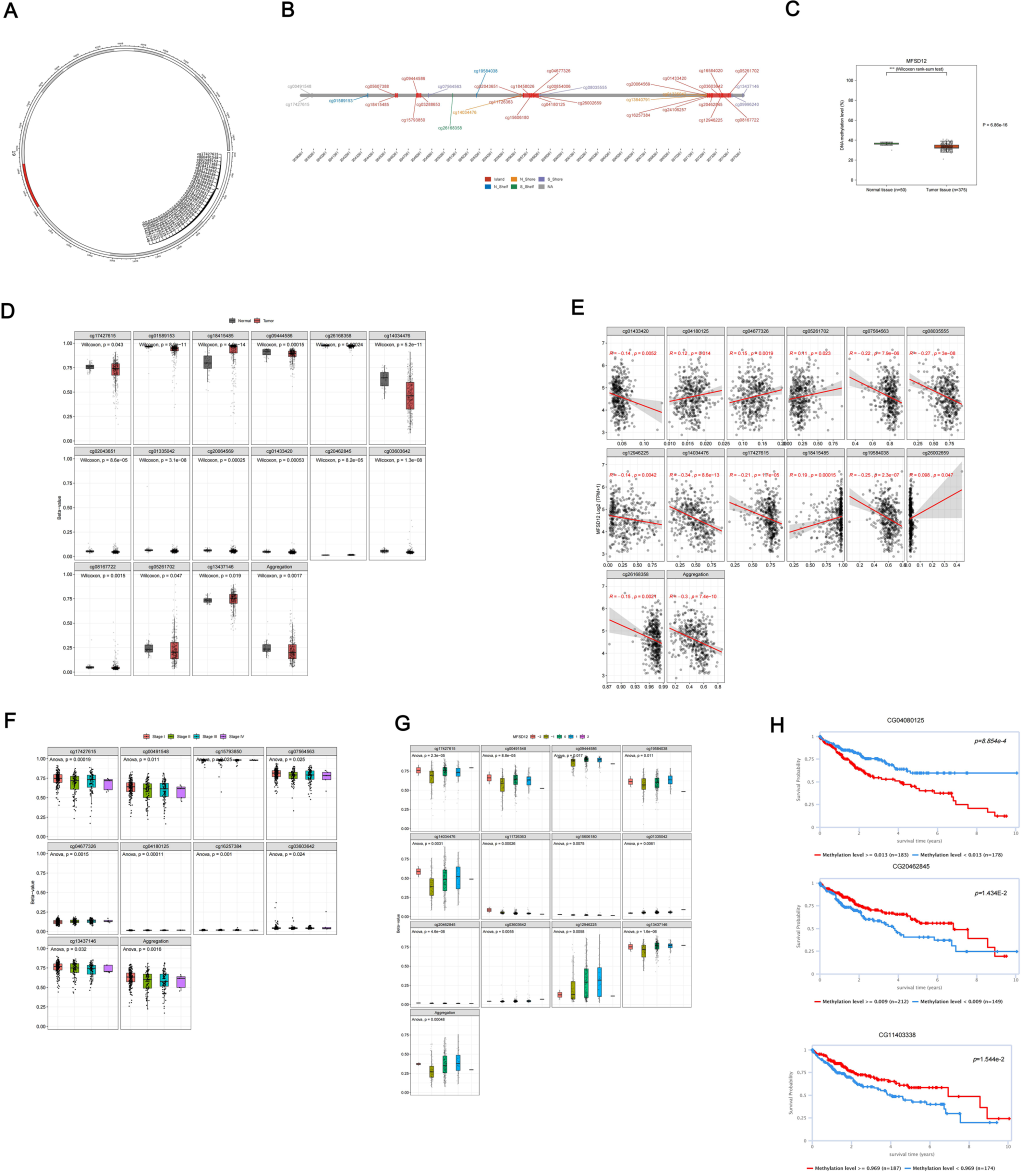
DNA methylation analysis of the MFSD12 genomic features in LIHC. **(A)** The chromosomal localization of MFSD12 within the human genome. **(B)** The genomic architecture of MFSD12 and its adjacent regions. **(C)** The dynamics of promoter methylation in LIHC and normal liver tissues. **(D)** MFSD12 methylation levels in tumor tissue samples compared to normal tissues. **(E)** Analysis of the correlation between MFSD12 expression and its methylation status. **(F)** The identification of tumor stage-specific methylation alterations. **(G)** The relationship between MFSD12 individual CpG site methylation values and CNV status (deep deletion, loss, neutral, gain, amplification). **(H)** The association of MFSD12 methylation with patient survival outcomes. ***P < 0.001. CpG, Cytosine-phosphate-Guanine dinucleotide; LIHC, Liver Hepatocellular Carcinoma; TCGA, The Cancer Genome Atlas; TNM, Tumor-Node-Metastasis staging system.

DNA methylation plays a crucial role in the process of LIHC. In this study, we conducted a comprehensive analysis of the methylation status of each site within the MFSD12 gene, examining the correlation between MFSD12 methylation status, transcriptional expression, and clinical characteristics using the EWAS Data Hub and SMART APP. We also assessed its prognostic significance for survival in affected individuals. Our findings revealed a total of 35 CpG methylation sites within the GCN1 region (**Figures 6A, B**), with significantly reduced MFSD12 methylation levels observed in tumor tissue samples compared to normal tissues (**Figure 6C**). This trend was evident in 15 individual CpG sites (cg17427615, cg01589153, cg18415485, etc.) (**Figure 6D**). Then we conducted an analysis of the correlation between MFSD12 expression and its methylation status. Among 12 individual CpG sites, cg01433420, cg07564563, cg08035555, cg12946225, cg14034476, cg17427615, cg19584038, and cg26168358 demonstrated a significant negative correlation between their methylation levels and MFSD12 expression. This strong negative correlation, particularly at sites within the promoter region (e.g., cg17427615), suggests that hypermethylation likely suppresses MFSD12 transcription, potentially by inhibiting the binding of activating transcription factors or by promoting a repressive chromatin state. Consequently, the widespread hypomethylation observed in LIHC tumors provides a plausible epigenetic mechanism for the upregulation of MFSD12 expression, contributing to its oncogenic role. In contrast, cg04180125, cg05261702, cg18415485, and cg26002659 exhibited a positive correlation between their methylation levels and MFSD12 expression. The aggregated methylation values were significantly negatively correlated with MFSD12 expression (**Figure 6E**). Furthermore, we analyzed DNA methylation levels at specific CpG sites within the MFSD12 gene across various stages of cancer (Stage I to Stage IV). We found that MFSD12 DNA methylation levels were highest in Stage I and decreased progressively, reaching their lowest in Stage IV (**Figure 6F**). This progressive loss of methylation with advancing disease stage further underscores the dynamic nature of MFSD12 epigenetic regulation during LIHC progression and suggests that demethylation may be associated with more aggressive tumor behavior. Additionally, we examined the relationship between MFSD12 individual CpG site methylation values and CNV. We discovered a significant positive relationship between the -2 (homozygous deletion) CNV and the methylation values of individual CpG sites within MFSD12(**Figure 6G**). Finally, we conducted an analysis of the correlation between each methylation site of MFSD12 and the prognosis of patients with LIHC. Our findings, presented in **Figure 6H**, identified one methylation site (CG04080125) associated with an unfavorable prognosis and two sites (CG20462845, CG11403338) associated with a favorable prognosis.

### Enrichment analysis of genes co−expressed with MFSD12 in LIHC

**Figure 7:**
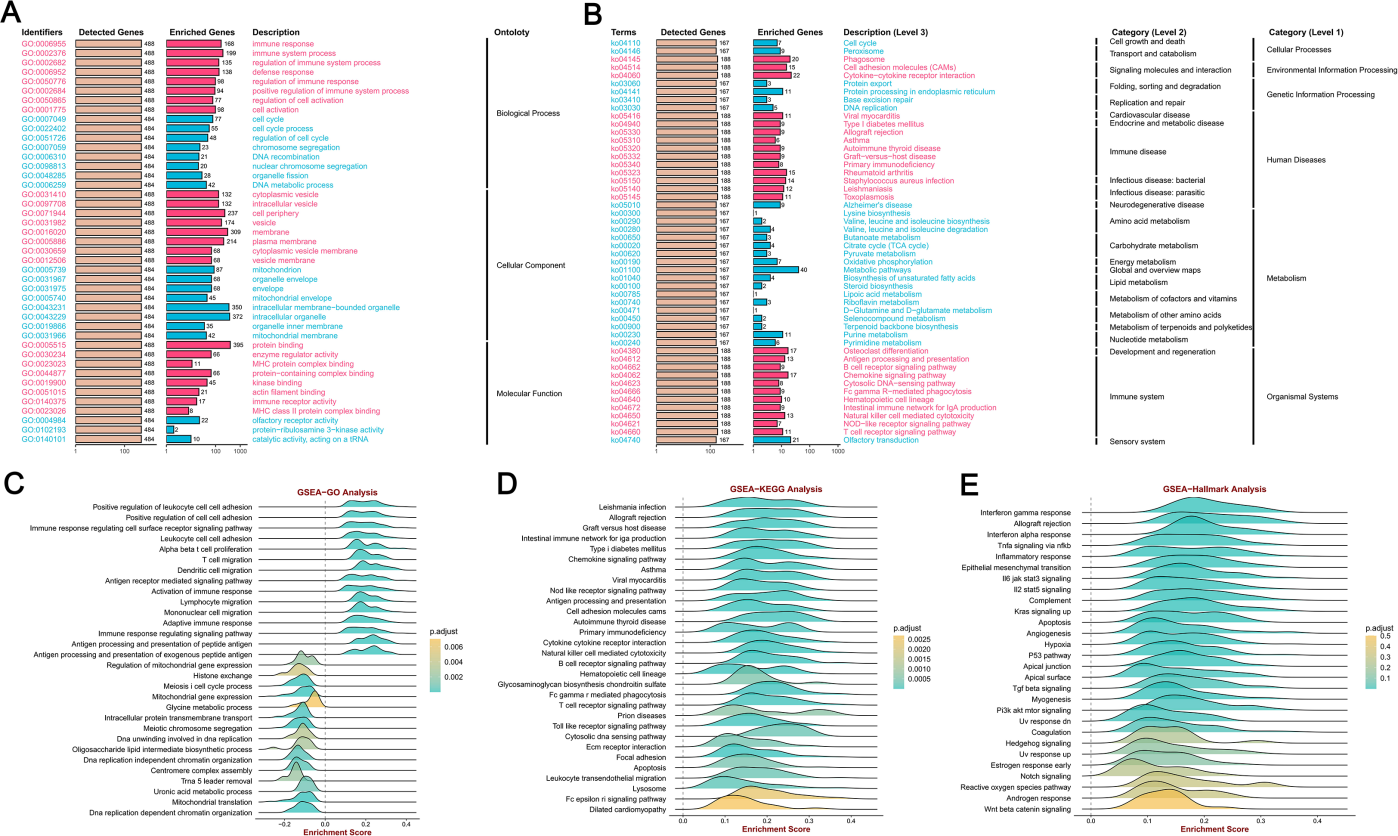
MFSD12 functional enrichment analysis across immune-related pathways and biological processes in LIHC. **(A)** GO enrichment analysis of MFSD12-associated biological processes. **(B)** KEGG pathway enrichment of MFSD12. **(C)** The GSEA-GO enrichment profile of MFSD12 in the context of immune regulation, as indicated by the enrichment score. **(D)** The GSEA-KEGG enrichment profile of MFSD12 in the context of immune regulation, as indicated by the enrichment score. **(E)** Hallmark gene set enrichment of MFSD12 in LIHC. ES, Enrichment Score; GSEA, Gene Set Enrichment Analysis; GO, Gene Ontology; KEGG, Kyoto Encyclopedia of Genes and Genomes; MFSD12, Major Facilitator Superfamily Domain Containing 12.

We employed GSEA tools to conduct KEGG pathway and GO analyses of MFSD12 using GO, HALLMARK, and KEGG gene sets. The GO analysis indicated significant enrichment of biological processes related to immune response and cell cycle regulation, including immune response, immune system process, regulation of immune system process, and defense response (**Figures 7A**). The cellular component (CC) analysis demonstrated that MFSD12 was associated with both intracellular and extracellular components, with enrichment in elements such as cytoplasmic vesicle and cell periphery. Furthermore, MFSD12 was linked to specific molecular functions (MF), with pathways enriched in functions such as protein binding and enzyme regulator activity. The GSEA-KEGG analysis revealed a strong positive enrichment signature in several pathways, notably including cytokine-cytokine receptor interaction, chemokine signaling pathway, osteoclast differentiation, phagosome, natural killer cell-mediated cytotoxicity, antigen processing and presentation, T cell receptor signaling pathway, and cell adhesion molecules (CAMs) (**Figures 7B**). The significant enrichment of immune-related pathways, particularly “cytokine-cytokine receptor interaction” and “chemokine signaling pathway”, was highly relevant to the LIHC microenvironment. These pathways were central to the recruitment and function of tumor-associated macrophages (TAMs) and other immunosuppressive cells, which were key players in LIHC progression and immunotherapy resistance. This suggested that MFSD12 might promote immunosuppressive TME by modulating these critical communication networks. Conversely, the negative enrichment in general metabolic pathways aligned with the metabolic reprogramming that was a hallmark of liver cancer. Simultaneously, a significant positive enrichment was observed in pathways associated with immune-disease-associated pathways, including rheumatoid arthritis, Staphylococcus aureus infection, leishmaniasis, toxoplasmosis, allograft rejection, and autoimmune thyroid disease. Conversely, a broad and pronounced negative enrichment was predominant in metabolic pathways, with the most substantial enrichment noted in general metabolic pathways. These findings suggested that MFSD12 was closely linked to immune activation and inflammatory processes within the LIHC microenvironment, while also being associated with a marked downregulation of core metabolic functions and DNA maintenance mechanisms. **Figures 7C, D** provided a detailed illustration of the Enrichment Scores from the GSEA-GO and GSEA-KEGG analyses, further corroborating the results presented in the aforementioned figure.

Moreover, the GSEA-Hallmark analysis revealed significant enrichment across a diverse array of pathways, as illustrated in **Figures 7E**. Pathways such as “Interferon gamma response”, “Allograft rejection” and “Inflammatory response’” demonstrated positive enrichment, indicating an upregulation of these pathways, which are essential for immune response and inflammation. In contrast, pathways such as “Wnt beta catenin signaling” and “Androgen response” exhibited negative enrichment, suggesting a downregulation that may reflect the inhibitory effects of MFSD12 on these signaling cascades. This comprehensive analysis highlighted the complex role of MFSD12 in LIHC, affecting both immune responses and cellular signaling, which could have implications for disease progression and potential therapeutic targets.

### The correlations between expression levels of MFSD12 and immune cell infiltration in LIHC

**Figure 8:**
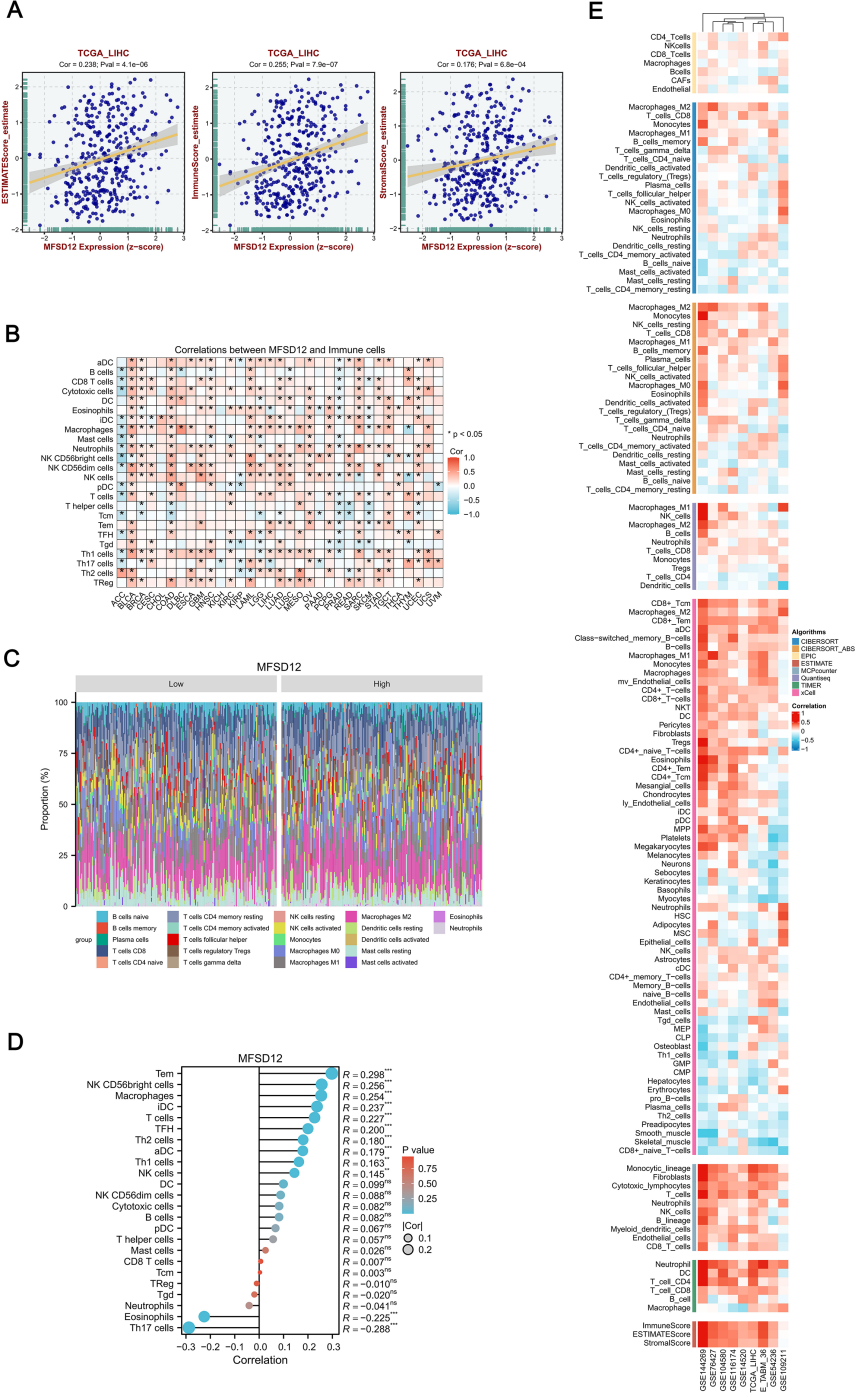
Integrative analysis of MFSD12 expression correlation with tumor microenvironment immunocytes in LIHC. **(A)** Correlation of MFSD12 with tumor microenvironment scores using algorithm of ESTIMATE database: association of MFSD12 with immune score, stromal score, and ESTIMATE score in LIHC. **(B)** Correlation of MFSD12 expression level with immune cell across 33 cancer types. **(C)** Comparison of immune cell proportions stratified by MFSD12 expression levels (Low vs. High) in TCGA_LIHC. **(D)** Relationship between MFSD12 expression and immune infiltration in LIHC, as analyzed by the ssGSEA algorithm. **(E)** Relationship between MFSD12 expression and immune infiltration in LIHC across a range of immune infiltration analysis tools and multiple genomic datasets. **P < 0.01, ***P < 0.001. CIBERSORT, cell-type identification by estimating relative subsets of RNA Transcripts; Cor, Pearson correlation coefficient; ESTIMATE, estimation of stromal and immune cells in malignant tumor tissues using expression data; LIHC, Liver Hepatocellular Carcinoma; Pval, p-value; TCGA, The Cancer Genome Atlas; xCell, cell type enrichment analysis tool.

In LIHC, the expression patterns of MFSD12 were significantly associated with clinical characteristics, whereas tumor-infiltrating lymphocytes serve as independent predictors of key clinical parameters, including tumor stage, grade, and lymph node status. The tumor microenvironment, comprising tumor cells, stromal cells, and immune infiltrating cells, plays a pivotal role in cancer progression. To further explore this relationship, we conducted an analysis using data from TCGA to assess the association between MFSD12 expression levels and immune cell infiltration in LIHC. Utilizing the “ESTIMATE” function within the R package, we examined the correlations between immune scores, estimate scores, stromal scores, and MFSD12 expression in LIHC. Our analysis demonstrated a positive correlation between MFSD12 expression and the estimate score (R = 0.238, P = 4.1e − 06), the immune score (R = 0.255, *P* = 7.9e - 07) and the stromal score (R = 0.176, *P* = 6.8e − 04), suggesting its potential involvement in augmenting stromal and immune activities (**Figure 8A**).

Next, we examined MFSD12 expression across 33 cancer types and its impact on 24 immune cell types, revealing distinct immunomodulatory patterns (**Figure 8B**). MFSD12 positively correlated with effector lymphocytes and myeloid cells, especially Tem cells (notably in THYM, KIRC, LIHC), macrophages (TGCT, UCEC, SKCM), and iDCs (LGG, PRAD, KICH). Conversely, it negatively correlated with immunosuppressive elements, particularly Th17 cells (UVM, DLBC, KIRC) and eosinophils (CHOL, THCA, STAD). Over 78% of cancer types showed significant associations with Tem cells, macrophages, and iDCs, while Th17 cells had pan-cancer inverse relationships. Tissue-specific trends were noted, with LIHC showing strong dual regulatory effects, whereas brain tumors GBM, LGG had weaker correlations. NK cell subsets varied by cancer type, with CD56bright cells positively correlating in BRCA and OV but negatively in COAD and LUSC. The persistent occurrence of asterisks underscored the universal role of MFSD12 in macrophage recruitment, as evidenced by its positive association in 31 out of 33 cancer types, and in the suppression of Th17 cells, with a negative correlation observed in 29 out of 33 cancers. This established MFSD12 as a conserved regulator of tumor-associated immunity. **Figure 8C** illustrated the comparison of immune cell proportions stratified by MFSD12 expression low and high levels in TCGA_LIHC. The analysis revealed that high MFSD12 expression was associated with increased proportions of M2 macrophages, monocytes, and CD8 T cells, while it correlated with decreased proportions of resting NK cells, naive B cells, and neutrophils.

Furthermore, we conducted a comprehensive analysis of immune cell infiltration using the single-sample Gene Set Enrichment Analysis (ssGSEA) algorithm to examine the associations between MFSD12 expression and 24 immune cell subtypes (**Figure 8D**). Our findings revealed a spectrum of relationships, ranging from strong positive to strong negative correlations. MFSD12 exhibited moderate positive correlations with effector memory T cells (Tem, R = 0.298, *P* < 0.001), macrophages (R = 0.256, *P* < 0.001), and immature dendritic cells (iDC, R = 0.254, *P* < 0.001), suggesting co-enrichment within the tumor microenvironment. Additional associations were noted with follicular helper T cells (TFH, R = 0.227, *P* < 0.001), Th2 cells (R = 0.200, *P* < 0.001), and activated dendritic cells (aDC, R = 0.180, *P* < 0.001). In contrast, moderate negative correlations were observed with Th17 cells (R = -0.288, *P* < 0.001) and eosinophils (R = -0.225, *P* < 0.001), indicating mutual exclusion. Non-significant associations (*P* > 0.05) were found with CD8+ T cells (R = 0.007), regulatory T cells (Treg, R = -0.010), and neutrophils (R = -0.041), underscoring the specificity of MFSD12’s immunomodulatory effects. To enhance the validation of our findings, we utilized a range of immune infiltration analysis tools, namely EPIC, ESTIMATE, TIMER, MCP-Counter, QuanTIseq and XCELL, across several genomic datasets, including GSE144269, GSE76427, GSE104580, GSE116174, GSE14520, TCGA_LIHC, E_TABM_36, GSE54236, and GSE109211. The application of these algorithms to diverse genomic datasets collectively demonstrated a robust concordance in characterizing the tumor immune microenvironment, thereby reinforcing the consistency of our observations across various computational frameworks (**Figure 8E**).

To assess the consistency of immune infiltration estimates derived from different computational methods, we calculated the Spearman correlation coefficients between results from the ssGSEA and CIBERSORT algorithms. As shown in **Supplementary Figure S6**, moderate to strong correlations were observed for several immune cell types, such as Neutrophils (R = 0.37) and Tregs (R = 0.38). This analysis confirms the reliability of our immune infiltration estimates despite methodological differences.

### Immune regulatory genes, and immune checkpoints analysis of MFSD12 in LIHC

**Figure 9:**
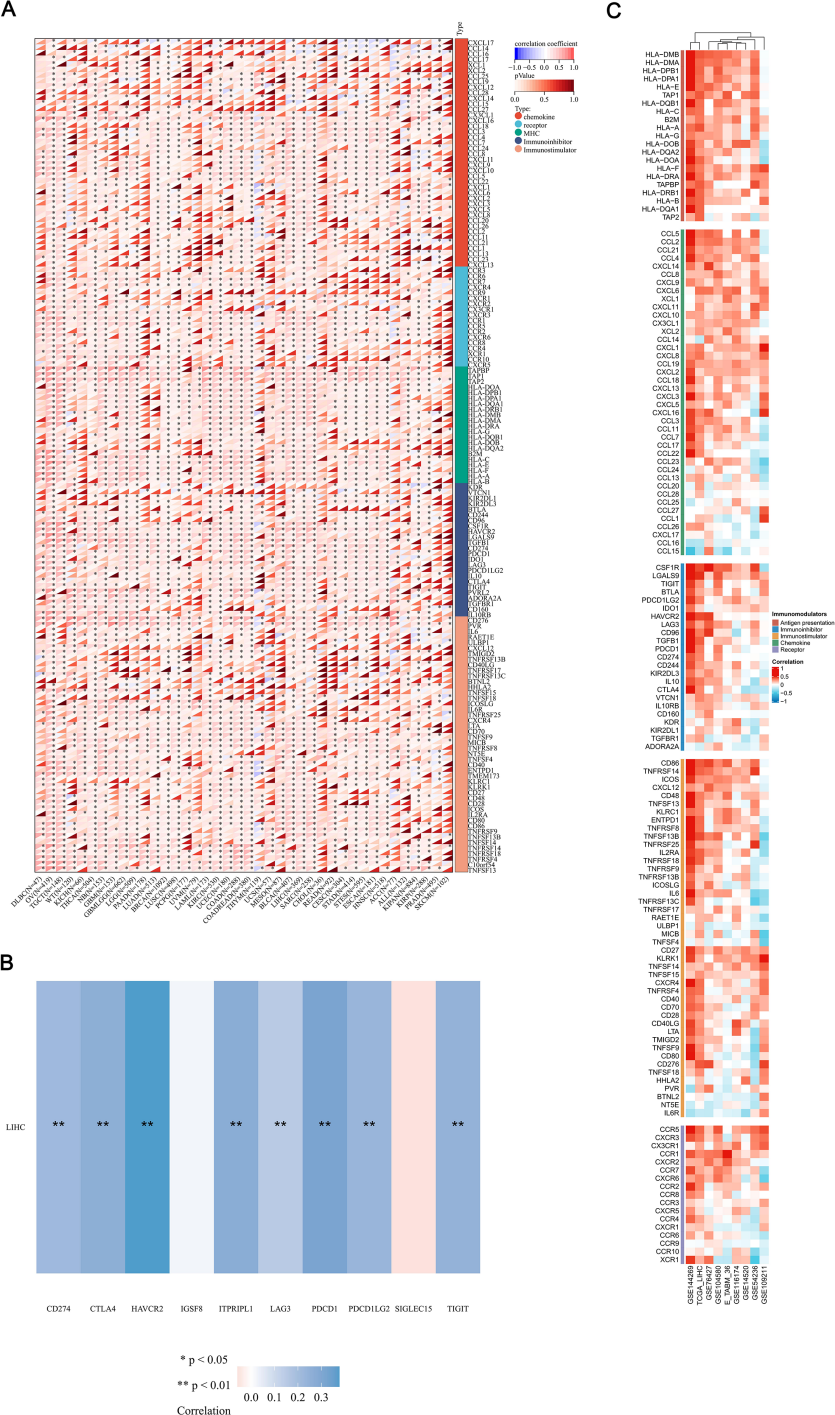
Integrated analysis of the link between MFSD12 expression and immune-related genes. **(A)** The relationship between the expression levels of MFSD12 and Immune-related genes in pan-cancers; **(B)** The relationship between the MFSD12 expression levels and immune checkpoints in LUSC. **(C)** Relationship between MFSD12 expression and Immune-related genes in LIHC across a range of immune infiltration analysis tools and multiple genomic datasets. *P < 0.05, **P < 0.01. LIHC, Liver Hepatocellular Carcinoma; Pearson, Pearson correlation coefficient; Cor, Correlation coefficient.

The effectiveness of immune checkpoint blockade (ICB) therapy is determined not only by the infiltration of immune cells but also by the presence of immune checkpoints and the expression of immune regulatory genes. In further continuation, we performed a comprehensive analysis of the correlations between the mRNA expression levels of MFSD12 and various immune-related genes, including chemokines, chemokine receptors, major histocompatibility complex (MHC) molecules, immunoinhibitors, and immunostimulators, across 32 different cancer types from TCGA (**Figure 9A**). MFSD12 demonstrated predominantly positive correlations across multiple cancer types, with distinct clusters indicating significant associations in specific cancers. Notably, in LIHC, the expression of MFSD12 was almost universally positively correlated with immune-related genes. Numerous transcripts related to immunological checkpoints, including SIGLEC15, PDCD1LG2 (PD-L2), TIGIT, PDCD1 (PD-1), CD274 (PD-L1), CTLA4, LAG3, and HAVCR2 (TIM3), are integral to tumor immune evasion mechanisms. Within the TCGA-LIHC cohort, the expression of MFSD12 demonstrated highly significant positive correlations with the transcript levels of these immune-checkpoint molecules (**Figure 9B**). Notably, HAVCR2 (TIM-3) exhibited the strongest association, suggesting a predominant co-regulation of this T-cell exhaustion marker. The B7/CD28 family inhibitors, PD-L1 (CD274) and PD-L2 (PDCD1LG2), displayed nearly equivalent correlations, which were mirrored by the immunoglobulin superfamily regulators TIGIT and LAG3. Additionally, associations were observed for CTLA4 and PD-1, whereas IGSF8 and SIGLEC15 showed non-significant correlations.

To substantiate our findings, we conducted a systematic analysis of the correlations between MFSD12 expression and 137 immune regulators across five functional categories: antigen presentation, chemokines, immunoinhibitors, immunostimulators, and receptors. This analysis was performed using nine independent cohorts, including TCGA-LIHC and GSE14520, among others (**Figure 9C**). The principal findings revealed a predominant positive correlation of immunosuppressive checkpoints, with HAVCR2 exhibiting the strongest correlation, followed by VTCN1 (B7-H4) and CD274 (PD-L1). Interestingly, LGALS9, the ligand of HAVCR2, showed a strong positive correlation with MFSD12. These results suggested a significant involvement in T-cell exhaustion pathways. Positive correlations were consistently observed with immune-related genes, thereby reinforcing the robustness of our observations. These findings implied that MFSD12 may play a pivotal role in modulating the tumor immune infiltration microenvironment in LIHC.

### Single-cell RNA sequencing analysis of MFSD12 expression

**Figure 10:**
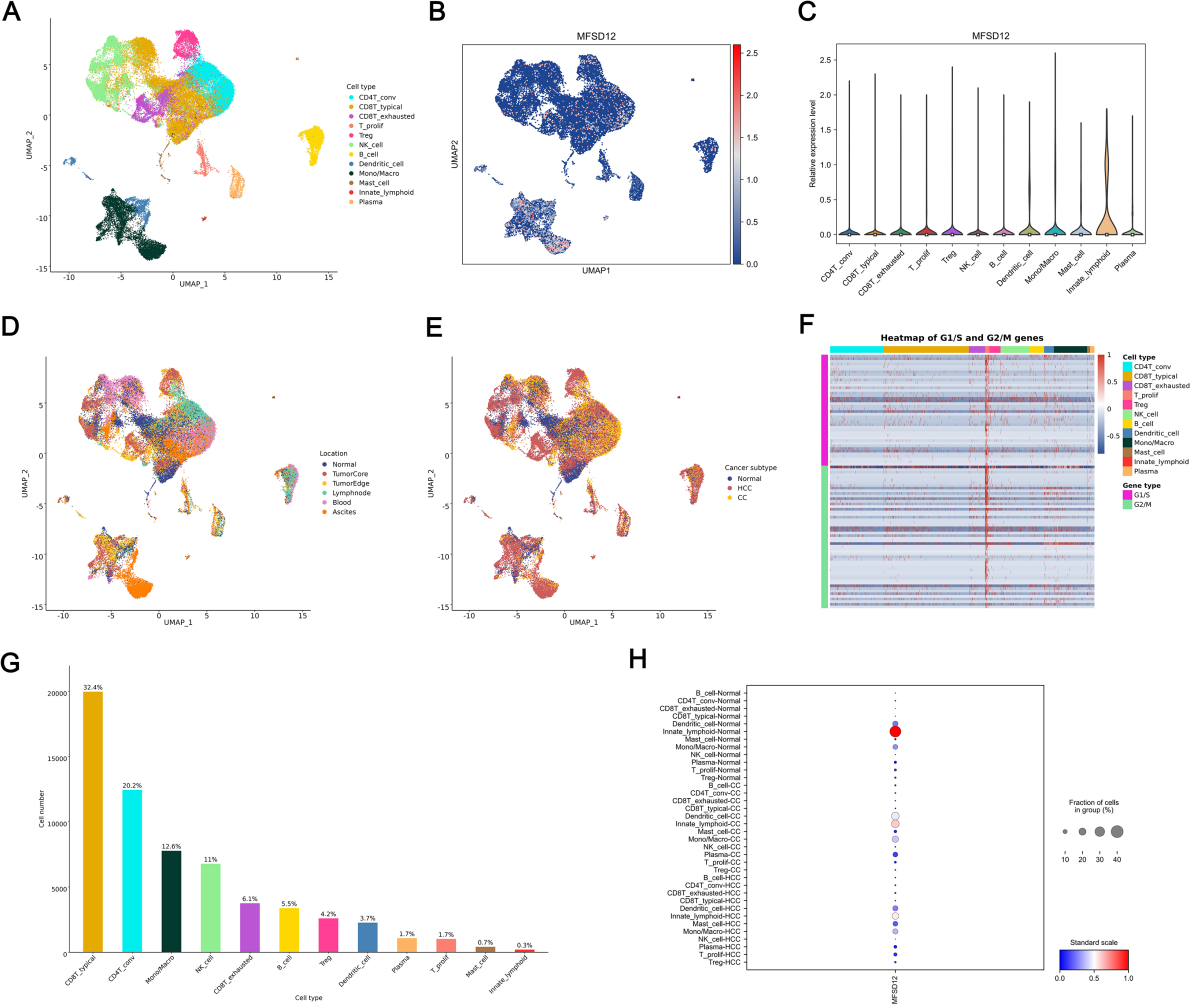
Single-Cell analysis of MFSD12 in LIHC by scRNA-seq. **(A)** UMAP visualization of cell type distribution in LIHC. **(B)** UMAP expression profile of MFSD12 in LIHC. **(C)** Relative expression levels of MFSD12 across cell types. **(D)** UMAP visualization of cell distribution by location. **(E)** UMAP visualization of cell distribution by cancer subtype (Normal, HCC, CC). **(F)** Heatmap of G1/S and G2/M phase transition gene expression across cell types. **(G)** Cell number and proportion statistics for each cell type. **(H)** Expression Proportion of MFSD12 in Different Cell Types and Cancer Subtypes. UMAP, Uniform Manifold Approximation and Projection; MFSD12, Major Facilitator Superfamily Domain Containing 12; CD4T_conv, Conventional CD4+ T cells; CD8T_typical, Typical CD8+ T cells; CD8T_exhausted, Exhausted CD8+ T cells; T_prolif, Proliferating T cells; Treg, Regulatory T cells; NK_cell, Natural Killer cell; B_cell, B lymphocyte; Mono/Macro, Monocyte/Macrophage; HCC, Hepatocellular Carcinoma; CC, Cholangiocarcinoma; G1/S, G1/S phase transition genes; G2/M, G2/M phase transition genes.

To investigate the cellular distribution and transcriptional regulation of MFSD12, we conducted single-cell RNA sequencing (scRNA-seq) analysis on LIHC tissues and adjacent normal liver tissue. The application of Uniform Manifold Approximation and Projection (UMAP) for dimensionality reduction revealed distinct clustering of major cell types, including CD4+ conventional T cells (CD4T_conv), exhausted CD8+ T cells (CD8T_exhausted), proliferating T cells (T_prolif), regulatory T cells (Treg), natural killer (NK) cells, B cells, dendritic cells (DCs), monocytes/macrophages (Mono/Macro), mast cells, innate lymphoid cells, and plasma cells (**Figure 10A**). Within the UMAP landscape, MFSD12 expression was spatially restricted, with discrete cell clusters exhibiting elevated transcript levels (**Figure 10B**). Quantitative analysis demonstrated that MFSD12 was most abundantly expressed in innate lymphoid cells (ILCs), monocytes/macrophages, DCs, mast cells, T_prolif, and Treg, followed by CD4+ conventional T cells and exhausted CD8+ T cells, with minimal expression observed in typical CD8+ T cells, B cells, and NK cells (**Figure 10C**). To enhance our understanding of the anatomical distribution of MFSD12-expressing cells, we mapped the identified clusters to their respective tissue origins, including the tumor core, tumor edge, adjacent normal tissue, and blood. Our findings indicated that cells with high MFSD12 expression were predominantly localized within the tumor core and edge, while exhibiting a sparse distribution in normal tissues and blood (**Figure 10D**). Within tumor subtypes, MFSD12 expression was notably enriched in LIHC compared to cholangiocarcinoma (CC) and normal controls, suggesting a subtype-specific regulatory mechanism (**Figure 10E**). A heatmap analysis of G1/S and G2/M phase marker genes revealed significant positive associations between MFSD12 expression and genes involved in the G1/S and G2/M phases within the T_prolif cell type (**Figure 10F**). Quantitative analysis of cell types confirmed that CD8+ typical T cells constituted the most abundant subset, followed by CD8+ exhausted T cells and monocytes/macrophages (**Figure 10G**). Finally, an examination of MFSD12 expression across immune cell types in normal, cholangiocarcinoma, and LIHC tissues demonstrated that innate lymphoid-normal cells exhibited the highest levels of MFSD12 expression, whereas other normal cells showed lower expression levels (**Figure 10H**). In CC and LIHC, Innate lymphoid-CC and Innate lymphoid-HCC cells exhibited elevated MFSD12 expression. Although the overall expression levels and cell fractions were higher than those observed in normal conditions, they differed across disease states, indicating the presence of context-dependent regulatory mechanisms. These findings underscored the cell-type-specific and condition-specific modulation of MFSD12, suggesting its potential involvement in the dynamics of immune response during carcinogenesis.

### Response to immunotherapy and drug sensitivity

**Figure 11:**
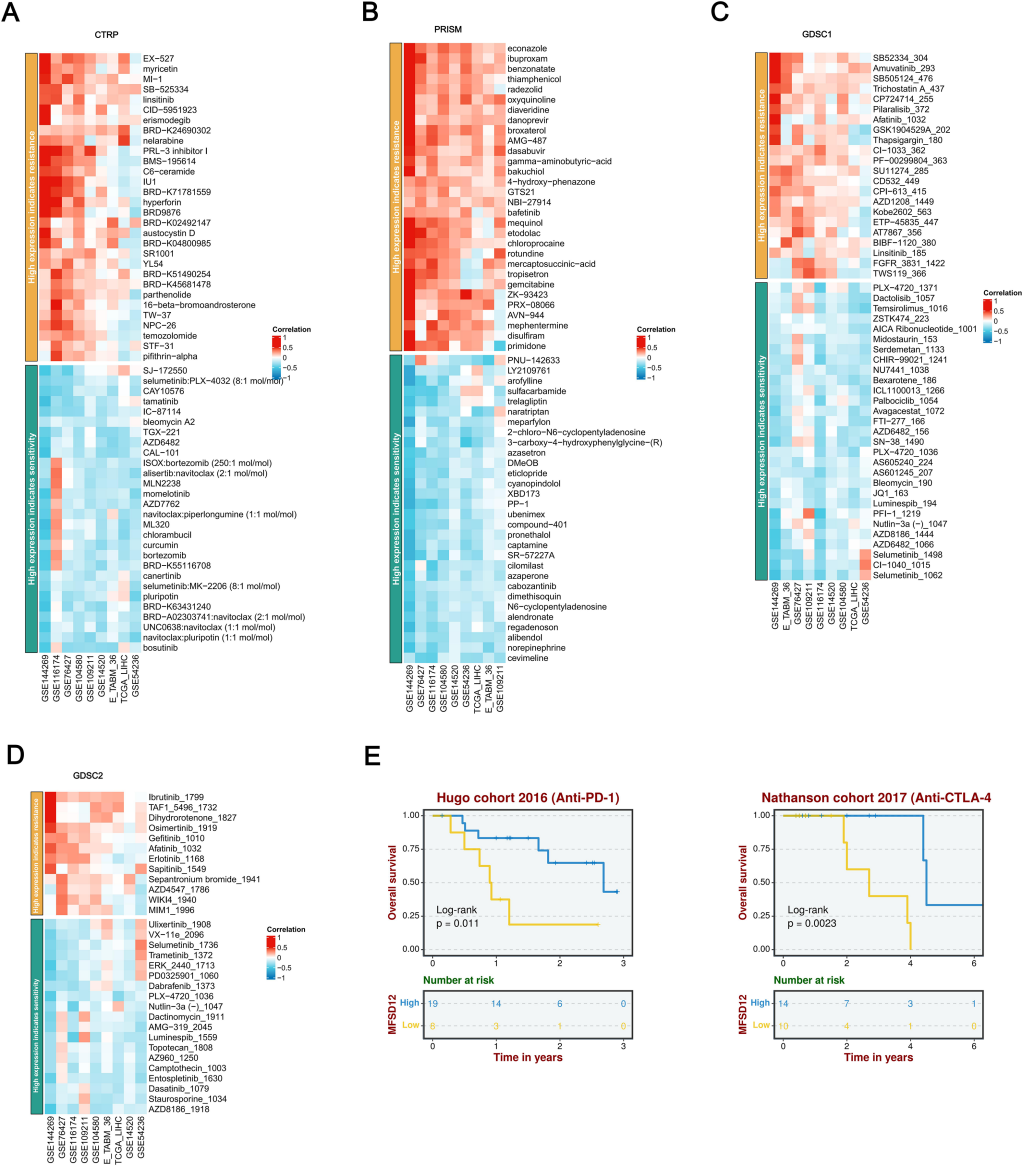
A thorough analysis of the correlation between MFSD12 expression and drug response across various databases, as well as its association with survival outcomes. **(A)** Correlation between MFSD12 expression and drug resistance/sensitivity in the CTRP dataset. **(B)** Correlation between MFSD12 expression and drug resistance/sensitivity in the PRISM dataset. **(C)** Correlation between MFSD12 expression and drug resistance/sensitivity in the GDSC1 database. **(D)** Correlation between MFSD12 expression and drug resistance/sensitivity in the GDSC2 database. **(E)** Overall survival analysis of Hugo cohort 2016 (Anti-PD-1) and Nathanson cohort 2017 (Anti-CTLA-4). CTRIP, Cancer Therapeutics Response Portal; PRISM, Preclinical Repurposing of Medicines; GDSC1/GDSC2, Genomics of Drug Sensitivity in Cancer 1/2; Anti-PD-1, Anti-Programmed Cell Death Protein 1; Anti-CTLA-4, Anti-Cytotoxic T-Lymphocyte-Associated Protein 4; Log-rank, Log-rank test; Number at risk, Number of patients at risk at each time point.

A comprehensive pharmacogenomic analysis conducted across a range of datasets (GSE144269, GSE76427, GSE116174, GSE104580, GSE14520, GSE54236, TCGA_LIHC, E_TABM_36, GSE109211) and drug screening platforms (CTRP, PRISM, GDSC1, GDSC2) demonstrated that increased expression of MFSD12 was consistently and significantly associated with resistance to a wide array of therapeutic agents (**Figures 11A-D**). Notably, this included resistance to EGFR inhibitors (such as Afatinib, Erlotinib, Gefitinib, Osimertinib, CI-1033), DNA-damaging agents (such as Sepantronium Bromide, Temozolomide), PI3K/mTOR inhibitors (such as Pilaralisib), BTK inhibitors (such as Ibrutinib), FGFR inhibitors (such as AZD4547), and WNT pathway inhibitors (such as WIKI4). Additionally, resistance was observed with various other compounds, including TAF1_5496, MIM1, gamma-aminobutyric acid (GABA), Bakuchiol, GTS21, NBI-27914, Bafetinib, Gemcitabine, Disulfiram, Econazole, Ibuproxam, Benzonatate, Thiamphenicol, and Radezolid, among others. In contrast, elevated MFSD12 expression exhibited a robust positive correlation, indicating increased sensitivity to a range of agents, including MEK/ERK pathway inhibitors (such as Selumetinib, Trametinib, Ulixertinib, VX-11e, PD0325901, CI-1040), DNA-damaging agents (including Dactinomycin, Topotecan, Camptothecin, Bleomycin), Bcl-2/BCR-ABL inhibitors (such as Navitoclax combinations, Bosutinib, Dasatinib), proteasome inhibitors (such as Bortezomib combinations), PI3K/mTOR inhibitors (including Dactolisib, Temsirolimus, ZSTK474), as well as other compounds like Cevimeline, Nutlin-3a, Luminespib, PLX-4720, Dabrafenib, Staurosporine, PNU-142633, Chlorambucil, Alisertib, and CAL-101. This highlighted a distinct and opposing pattern of drug response dependent on MFSD12 expression levels, consistently observed across all analytical platforms. Survival analysis within the Hugo cohort (2016, anti–PD-1 therapy) revealed that patients undergoing anti-PD-1 immunotherapy with high MFSD12 expression experienced significantly better OS compared to those with low expression (**Figure 11E**). Similarly, in the Nathanson cohort (2017, anti–CTLA-4 therapy), patients receiving anti-CTLA-4 immunotherapy with high MFSD12 expression were associated with a favorable prognosis, further confirming MFSD12’s role as a potential predictor of immunotherapy response.

### MFSD12 promotes the proliferation, migration, and invasion of LIHC cells

**Figure 12:**
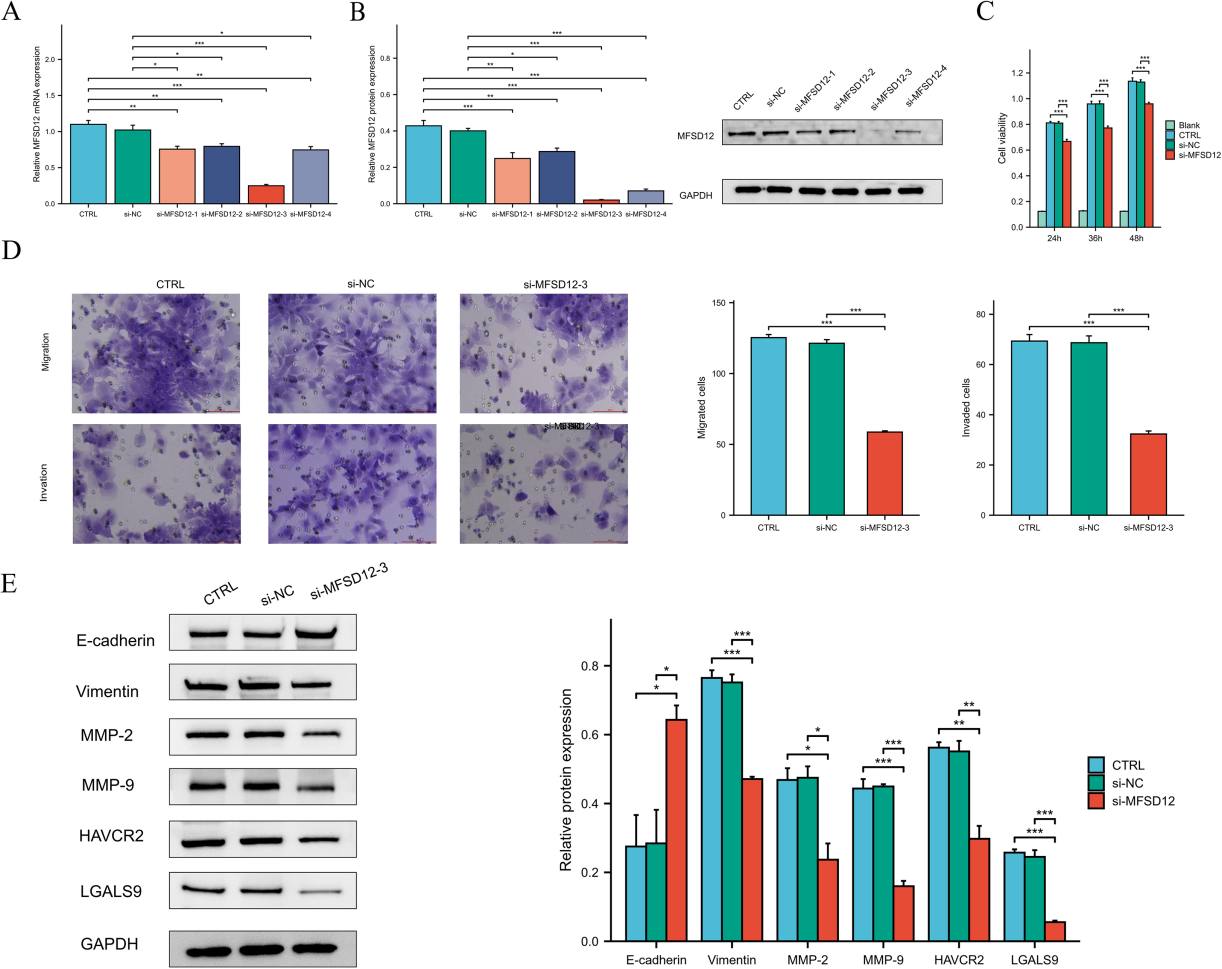
The knockdown of MFSD12 inhibited the proliferation, migration, and invasion of LIHC cells, as well as the TIM-3/Galectin-9 signaling pathway. **(A, B)** RT-qPCR and Western blot validation of MFSD12 silencing efficiency using siRNAs (si-MFSD12–1 to −4) with GAPDH as loading control. **(C)** CCK-8 cell viability assay showing reduced HEP 3B2.1–7 cells proliferation after MFSD12 knockdown (si-MFSD12-3). **(D)** Transwell assay revealed a reduction in the migratory and invasive capabilities of HEP 3B2.1–7 cells following the knockdown of MFSD12. **(E)** Immunoblot analysis of EMT markers and TIM-3 axis components showing up-regulation of E-cadherin and down-regulation of Vimentin, MMP-2, MMP-9, HAVCR2 (TIM-3) and LGALS9 in si-MFSD12-treated cells. *P < 0.05, **P < 0.01, ***P < 0.001. CTRL, control untreated; si-NC, negative control siRNA; si-MFSD12, MFSD12-targeting siRNA; E-cadherin, epithelial cadherin; MMP-2/9, matrix metalloproteinase-2/9; HAVCR2, hepatitis A virus cellular receptor 2 (TIM-3); LGALS9, lectin galactoside-binding soluble 9 (Galectin-9).

To elucidate the functional role of MFSD12 in the progression of hepatocellular carcinoma, we conducted *in vitro* experiments utilizing HEP 3B2.1–7 cells subjected to MFSD12 knockdown. Four distinct siRNA constructs targeting MFSD12 were transfected into these cells, and the successful downregulation of MFSD12 expression was confirmed via western blot and RT-qPCR analyses, as compared to negative control groups (**Figures 12A,B**). The most efficacious siRNA sequence(si-MFSD12-3) was subsequently selected for further functional assays. Proliferation analysis using the CCK-8 assay demonstrated that the depletion of MFSD12 significantly impaired the growth potential of HEP 3B2.1–7 cells (**Figure 12C**). Furthermore, MFSD12 knockdown markedly reduced the migration and invasion capabilities of the HEP 3B2.1–7 cell line, as evidenced by transwell migration and invasion assays (**Figure 12D**). Epithelial-mesenchymal transition (EMT) is a critical process for cancer cell invasion and migration. During EMT in tumor cells, the expression of proteins that promote cell-cell adhesion, such as E-cadherin, is decreased, while the expression of mesenchymal markers, including vimentin, MMP-2, and MMP-9, is increased, thereby enhancing cell migration and invasion capabilities (48–50). Subsequently, we investigated these EMT markers due to their known association with cellular invasion and migration. Our analysis of protein expression revealed that MFSD12 knockdown resulted in a significant upregulation of E-cadherin, accompanied by a concurrent downregulation of vimentin, MMP-2, and MMP-9 (**Figure 12E**). These findings offered compelling evidence that MFSD12 was integral to enhancing the proliferative and metastatic potential of liver cancer cells through the modulation of EMT-related pathways.

### MFSD12-siRNA decreased HAVCR2 and iLGALS9 expression in LIHC cells

In our investigation into the relationship between MFSD12 and immune cell infiltration, we identified a predominantly positive correlation between MFSD12 and some immunosuppressive checkpoints. Notably, a robust association was observed between MFSD12 expression and the expression levels of HAVCR2 as well as its ligand galectin-9 (LGALS9). To further explore this relationship, we conducted *in vitro* experiments using HEP 3B2.1–7 cells with MFSD12 knockdown. Our findings demonstrated that the downregulation of MFSD12 expression resulted in a significant decrease in the expression levels of HAVCR2 and LGALS9 in LIHC cells (**Figure 12E**). These results strongly suggested that MFSD12 might play a critical regulatory role in modulating the expression of HAVCR2 and LGALS9.

## Discussion

MFSD12 is a transmembrane protein vital for importing cysteine into melanosomes and lysosomes, maintaining normal cystine levels. It is crucial for pigmentation by aiding pheomelanin synthesis via cysteinyldopa production in melanosomes (22). Recent research has broadened the understanding of MFSD12’s involvement in oncological biology. This protein has been identified as a critical factor in the proliferation and progression of various malignancies, including melanoma, breast cancer, and lung cancer (26). Its function as a cysteine transporter implies that MFSD12 may influence tumor development and metastasis by affecting cellular redox states and metabolic pathways. In the context of LIHC, investigations into MFSD12’s role remain in the early stages. Nonetheless, its involvement in cysteine transport and broader implications in cancer biology suggest that it could play a pivotal role in the pathogenesis of LIHC. Given the liver’s central role in metabolic and detoxification processes, it represents a crucial site for examining the effects of dysregulated cysteine transport. Moreover, the potential association between MFSD12 and lysosomal storage disorders may offer insights into novel mechanisms underlying LIHC development, particularly in scenarios where metabolic and storage pathways are compromised.

Our research found that MFSD12 mRNA expression was significantly higher in most cancer tissues compared to normal ones. In LIHC, this overexpression was confirmed using TCGA-LIHC data and the combined TCGA and GTEx dataset. Overexpression was more common in males and females separately. Additionally, higher MFSD12 levels were linked to lower AFP levels and a history of alcohol consumption. The HPA database and immunohistochemical analysis also revealed significantly higher MFSD12 expression in LIHC tissues compared to adjacent non-tumor tissues. Subsequently, we found that high MFSD12 expression in LIHC was associated with poorer clinical outcomes. Univariate and multivariate Cox regression analyses identified high MFSD12 expression as a significant risk factor for OS, even after adjusting for pathologic T stage, AFP level, and age, with pathologic T stage being an independent prognostic factor. Gene Mutation Analysis indicated that MFSD12’s prognostic role in LIHC was primarily expression-driven rather than mutation-dependent. GSEA analyses showed that MFSD12 was positively enriched in immune-inflammatory pathways, such as cytokine interactions and T cell signaling, but negatively enriched in core metabolic pathways like DNA repair, suggesting it boosts immune activation while inhibiting metabolic balance in LIHC. Hallmark analysis indicated that MFSD12 upregulates interferon-γ response and inflammation, while downregulating Wnt/β-catenin signaling and androgen response, acting as both a promoter of immune pathways and a suppressor of cancer-related processes in the LIHC environment. This finding comprehensively revealed the comprehensive landscape of MFSD12 in LIHC and was consistent with existing literature that underscores the oncogenic function of MFSD12 across multiple cancer types. In LIHC, the upregulation of MFSD12 might disrupt the normal regulation of the cell cycle, allowing cancer cells to divide more rapidly and evade apoptosis.

When compared to its role in other malignancies such as melanoma and breast cancer, where MFSD12 primarily drives tumorigenesis through metabolic reprogramming and proliferation, our study unveils a distinctive facet of MFSD12 in LIHC. Beyond its cell-autonomous oncogenic functions, we identified a prominent role for MFSD12 in sculpting the immune landscape, particularly through its strong correlation with the HAVCR2/LGALS9 checkpoint axis. This immune-regulatory function appears to be more pronounced in LIHC, potentially reflecting the unique immunological context of the liver.

The tumor immune microenvironment has been shown to influence tumor growth, invasion, and metastasis. Understanding the composition of immune cells within tumor tissues could aid in the development of innovative therapeutic strategies and enhance the effectiveness of ICB therapy. Given that LIHC demonstrates relatively low immunogenicity and suboptimal responses to immunotherapy, we examined the link between immune-related scores and MFSD12 expression in LIHC, finding a positive correlation between MFSD12 expression and the estimate, immune, and stromal scores. This suggested MFSD12 might enhance stromal and immune activities. The immune score indicates immune cell presence and activity in the TME, with higher scores linked to better responses to neoadjuvant chemoradiotherapy and improved survival. Stromal scores can identify therapy targets and predict outcomes. Estimate scores combine stromal and immune scores to assess the TME, with low scores (high tumor purity) linked to aggressive behavior and immunotherapy resistance (51, 52). MFSD12 might support tumor cell independence, reduce stromal reliance and increase purity. We hypothesized that abnormal MFSD12 expression could impact immune engagement. Furthermore, we demonstrated that elevated MFSD12 expression was associated with increased proportions of M2 macrophages, monocytes, and CD8+ T cells, while it was correlated with decreased proportions of resting NK cells, naive B cells, and neutrophils. This relationship highlighted the intricate interaction between MFSD12 and the immune system, which was essential for understanding its role in cancer biology and its potential therapeutic implications. The findings supported the hypothesis that MFSD12 might enhance the infiltration and activity of CD8+ T cells and macrophages, thereby contributing to its role in the tumor microenvironment. Conversely, the negative correlation suggested that MFSD12 might lead to a diminished presence of cytotoxic and inflammatory cells within the tumor microenvironment, potentially facilitating tumor immune evasion and progression.

Our study identified a robust positive correlation between MFSD12 expression and transcripts of immune checkpoint molecules, notably HAVCR2(TIM3), suggesting potential co-regulation with this marker of T-cell exhaustion. Additionally, LGALS9, the ligand for HAVCR2, demonstrated a strong positive correlation with MFSD12. Similar correlation patterns were observed with PD-L1 (CD274) and PD-L2 (PDCD1LG2) from the B7/CD28 family, as well as TIGIT and LAG3 from the immunoglobulin superfamily. The interaction between HAVCR2 and its ligand, LGALS9, has been identified as a critical axis in the regulation of immune responses within oncological contexts. This pathway plays a pivotal role in modulating immune tolerance and facilitating immune evasion mechanisms across various malignancies. The TIM3/LGALS9 interaction is recognized for its immunosuppressive effects, which tumors can exploit to circumvent immune surveillance, thereby promoting tumor progression and correlating with poor prognosis in cancer patients (53, 54). Subsequently, we conducted *in vitro* experiments utilizing HEP 3B2.1–7 cells with targeted knockdown of MFSD12. Our findings indicated that the downregulation of MFSD12 expression resulted in a marked decrease in the expression levels of HAVCR2 and LGALS9 in LIHC cells. These results implied that MFSD12 might serve a pivotal regulatory function in modulating the expression of HAVCR2 and LGALS9. Furthermore, it remained to be elucidated whether MFSD12 knockdown impeded the binding of TIM3 to its ligand and whether it mitigated the immunosuppressive effect induced by the TIM3-ligand interaction-mediated T cell apoptosis. These questions merited further investigation.

scRNA-seq showed that MFSD12 was mainly expressed in ILCs, monocytes/macrophages, dendritic cells, mast cells, T_prolif, and Tregs in LIHC, with low expression in CD8+ T cells, B cells, and NK cells. These MFSD12-expressing cells were significantly more abundant in the LIHC tumor core and edge than in nearby normal tissue, blood, or cholangiocarcinoma, indicating subtype-specific upregulation. Pharmacogenomic analysis showed that high MFSD12 expression was linked to resistance against EGFR, BTK, FGFR, and WNT inhibitors but sensitivity to MEK/ERK inhibitors, certain DNA-damaging agents, Bcl-2/BCR-ABL inhibitors, and proteasome inhibitors. This suggested MFSD12 predicted a unique drug response pattern, indicating resistance to some drugs and sensitivity to others across various platforms.

Given the correlation between dysregulation of MFSD12 expression and adverse clinical outcomes in LIHC, it is crucial to elucidate the molecular functions of MFSD12. To address this gap in knowledge, we conducted comprehensive *in vitro* studies to characterize the role of MFSD12 in LIHC cell proliferation and metastatic behavior. Initially, we developed specific siRNA constructs to knock down MFSD12 expression in the HEP 3B2.1–7 cell line. Functional assays indicated that MFSD12 knockdown significantly impaired cellular proliferation, as demonstrated by CCK-8 viability assays. Furthermore, transwell experiments showed a substantial reduction in the migratory and invasive capacities of MFSD12-deficient cells compared to controls. Considering the pivotal role of EMT in tumor progression, we conducted an investigation into the principal molecular mediators involved in this process. EMT is typically characterized by the suppression of epithelial markers such as E-cadherin and the induction of mesenchymal proteins, including vimentin and MMP-2/9. Western blot analysis demonstrated that the silencing of MFSD12 led to a significant increase in E-cadherin levels, accompanied by a reduction in the expression of vimentin, MMP-2, and MMP-9. These findings strongly indicated that MFSD12 might play a role in the pathogenesis of LIHC by promoting tumor cell proliferation and facilitating EMT-mediated metastasis. Nonetheless, the specific molecular pathways through which MFSD12 exerts these oncogenic effects have yet to be fully elucidated.

From a therapeutic perspective, our findings nominated MFSD12 as a candidate for targeted intervention in LIHC. Given its structure as a member of the Major Facilitator Superfamily, future efforts could focus on developing small-molecule inhibitors that targeted the conserved MFS_2 transporter domain of MFSD12. Such inhibitors could potentially disrupt its function in cysteine transport and immune modulation, offering a novel combinatorial strategy with existing immune checkpoint blockers.

This study had several limitations that should be acknowledged. First, our functional *in vitro* experiments were primarily conducted in a single LIHC cell line (HEP3B2.1-7), and future studies should validate these findings in a broader panel of cell lines. Second, the lack of *in vivo* validation using animal models meant the physiological relevance of MFSD12’s role in LIHC progression and immune modulation required further confirmation. Third, although our multivariate Cox model included major clinical parameters, the lack of universally available data on liver cirrhosis status and HBV/HCV infection in the TCGA-LIHC cohort was a limitation. Future studies with more comprehensively annotated clinical datasets were needed to fully ascertain the independent prognostic value of MFSD12 after adjusting for these crucial liver-specific factors. Finally, the precise molecular mechanism by which MFSD12 influences the HAVCR2/LGALS9 axis remained to be fully elucidated.

In conclusion, our comprehensive study elucidated the multifaceted roles of MFSD12 in the progression of LIHC and its impact on immune modulation. We observed that increased expression of MFSD12 promoted tumor growth while creating an immune-evasive microenvironment by affecting immune cell recruitment and the production of immunosuppressive factors. Targeted inhibition of MFSD12 demonstrated significant therapeutic potential by concurrently reducing malignant cell proliferation and invasion, and enhancing the efficacy of immune checkpoint blockade, particularly when used in conjunction with TIM3 inhibition. These findings underscored the potential of MFSD12 as a prognostic biomarker and a promising molecular target for LIHC treatment. Incorporating MFSD12 profiling into clinical practice could enable personalized therapeutic strategies and improve survival outcomes for patients.

## Data Availability

The data presented in the study are retrieved from public repositories, including the TCGA repository (https://portal.gdc.cancer.gov/, accession: TCGA-LIHC), GTEx repository (http://gtexportal.org/), GEO repository (https://www.ncbi.nlm.nih.gov/geo/, accessions: GSE144269, GSE76427, GSE104580, GSE116174, GSE14520, GSE54236, GSE109211), and ArrayExpress repository (https://www.ebi.ac.uk/arrayexpress/, accession: E_TABM_36).

## Supplementary material

Supplementary Figure 1 MFSD12 expression levels before (Pre) and after (Post) treatment in LIHC patients, stratified by gender. Analysis was performed using the GSE196434 dataset. The observed differential expression patterns following treatment, which vary between male and female patients, may contribute to the divergent gender-associated expression trends identified in the TCGA-LIHC and GSE76427 cohorts, potentially reflecting differences in baseline clinical characteristics and treatment status.

Supplementary Figure 2 MFSD12 expression is significantly altered following intervention in LIHC. MFSD12 transcript levels were measured before (Pre) and after (Post) a specific intervention in the GSE213797 dataset. Expression values are presented as log2(TPM + 1). Statistical significance is indicated as follows: *p < 0.05; **p < 0.01; ***p < 0.001; ns, not significant. This result reinforces the dynamic regulation of MFSD12 in LIHC-related conditions.

Supplementary Figure 3 Time-dependent receiver operating characteristic (ROC) analysis of MFSD12 for predicting survival in LIHC patients across multiple cohorts. ROC curves and corresponding area under the curve (AUC) values at 1, 2, 3, 4, and 5 years for MFSD12 expression are shown for the TCGA-LIHC cohort and four independent GEO cohorts (GSE116174, GSE144269, GSE14520, and GSE76427). The analysis demonstrates the predictive accuracy of MFSD12 for patient survival at different time points across diverse datasets.

Supplementary Figure 4 Bootstrap validation of the prognostic value of MFSD12 expression in LIHC across independent cohorts. Hazard ratios (HRs) for overall survival based on MFSD12 expression were calculated from 1,000 bootstrap samples in each of the five cohorts (TCGA-LIHC, GSE116174, GSE144269, GSE14520, and GSE76427). The stability of the HRs across resampled datasets confirms the robustness of MFSD12 as a prognostic factor in LIHC.

Supplementary Figure 5 Kaplan-Meier survival analysis of MFSD12 expression in LIHC patients across multiple independent cohorts. Overall survival curves for patients with high versus low MFSD12 expression are shown for the TCGA-LIHC cohort and four GEO cohorts (GSE116174, GSE144269, GSE14520, and GSE76427). Log-rank test p-values are indicated for each cohort. Although the statistical significance varies, a consistent trend towards poorer survival in patients with high MFSD12 expression is observed across all datasets, further supporting its prognostic value in LIHC.

Supplementary Figure 6 Correlation of immune cell infiltration estimates between ssGSEA and CIBERSORT algorithms. Spearman correlation coefficients (ρ) are shown for selected immune cell types, comparing estimates derived from the ssGSEA and CIBERSORT computational methods. Moderate to strong correlations were observed for several cell types, including Neutrophils (ρ=0.37) and regulatory T cells (Tregs, ρ=0.38), supporting the consistency and reliability of the immune infiltration analyses used in this study despite methodological differences.

Supplementary Table 1 Summary of sample information for the single-cell RNA sequencing cohort (GSE140228). The table provides the GEO sample accession numbers, sample source, and key characteristics for each specimen, including patient identifier, tissue origin (e.g., tumor, adjacent liver, blood, ascites, lymph node), and cell type (CD45+ immune cells). This metadata describes the cellular heterogeneity and tissue context of the 41 LIHC specimens analyzed in the single-cell RNA sequencing study.

Supplementary Table 2 Clinical characteristics of patients with LIHC. LIHC, liver hepatocellular carcinoma.

## Glossary

MFSD12: Major Facilitator Superfamily Domain-containing 12
LIHC: liver hepatocellular carcinoma
AFP: Alpha-fetoprotein
TCGA: The Cancer Genome Atlas
GEO: Gene Expression Omnibus
IACR: International Agency for Research on Cancer
IHC: immunohistochemistry
HPA: Human Protein Atlas
AUC: Area Under Curve
CI: Confidence Interval
ROC: Receiver Operating Characteristic
TPM: Transcripts Per Million
CC: Cholangiocarcinoma
CNV: Copy Number Variation
SNV: Single Nucleotide Variant
WES: Whole Exome Sequencing
CpG: Cytosine-phosphate-Guanine dinucleotide
TNM: Tumor-Node-Metastasis staging system
GSEA: Gene Set Enrichment Analysis
CIBERSORT: cell-type identification by estimating relative subsets of RNA Transcripts
Cor: Pearson correlation coefficient
ESTIMATE: estimation of stromal and immune cells in malignant tumor tissues using expression data
xCell: cell type enrichment analysis tool
Pearson: Pearson correlation coefficient
Cor: Correlation coefficient
UMAP: Uniform Manifold Approximation and Projection
MFSD12: Major Facilitator Superfamily Domain Containing 12
CD4T_conv: Conventional CD4+ T cells
CD8T_typical: Typical CD8+ T cells
CD8T_exhausted: Exhausted CD8+ T cells
T_prolif: Proliferating T cells
Treg: Regulatory T cells
NK_cell: Natural Killer cell
B_cell: B lymphocyte
Mono/Macro: Monocyte/Macrophage
HCC: Hepatocellular Carcinoma
CC: Cholangiocarcinoma
G1/S: G1/S phase transition genes
G2/M: G2/M phase transition genes
CTRP: Cancer Therapeutics Response Portal
PRISM: Preclinical Repurposing of Medicines
GDSC1/GDSC2: Genomics of Drug Sensitivity in Cancer 1/2
Anti-PD-1: Anti-Programmed Cell Death Protein 1
Anti-CTLA-4: Anti-Cytotoxic T-Lymphocyte-Associated Protein 4
Log-rank: Log-rank test
Number at risk: Number of patients at risk at each time point
CTRL: control untreated
si-NC: negative control siRNA
si-MFSD12: MFSD12-targeting siRNA
E-cadherin: epithelial cadherin
MMP-2/9: matrix metalloproteinase-2/9
HAVCR2: hepatitis A virus cellular receptor 2 (TIM-3)
LGALS9: lectin galactoside-binding soluble 9 (Galectin-9)
LUAD: Lung adenocarcinoma
LUSC: Lung squamous cell carcinoma
TMB: Tumor mutation burden;MSI, Microsatellite instability
GTEx: Genotype-Tissue Expression databases
OS: overall survival
PFS: progression-free survival
ROC: receiver operating characteristic
AUCs: area under the curves
C-index: consistency index
KM plotter: Kaplan-Meier plotter
IOD: integrated optical density
FC: fold-change
HR: Hazard ratio
siRNA: small interfering RNA
OD: optical density
GBM: Glioblastoma
GBMLGG: Glioblastoma and Lower Grade Glioma
LGG: Lower Grade Glioma
BRCA: Breast Cancer
KIRP: Kidney Papillary Cell Carcinoma
STAD: Stomach Adenocarcinoma
HNSC: Head and Neck Squamous Cell Carcinoma
KIRC: Kidney Renal Clear Cell Carcinoma
LIHC: Liver Hepatocellular Carcinoma
PAAD: Pancreatic Adenocarcinoma
TILs: tumor-infiltrating lymphocytes
TME: tumor microenvironment
ICI: Immune checkpoint inhibitors
