# Supplementary material for: MFSD12 promotes proliferation, metastasis and invasion of hepatocellular carcinoma cells and its potential correlation with HAVCR2/LGALS9 immune checkpoint axis
Source: Front Immunol. 2025 Oct 21;16:1681887. doi: 10.3389/fimmu.2025.1681887 (PMC12582970; doi:10.3389/fimmu.2025.1681887)
Supplement: Supplementary Table 2 — Clinical characteristics of patients with LIHC. LIHC, liver hepatocellular carcinoma. [file Table2.docx]

**Table S2** Clinical characteristics of patients with LIHC.

| Characteristic | No.of patients(%) |
| --- | --- |
| **n** | **19** |
| **Age, n (%)** |  |
| ≤55 | 13(68.4%) |
| >55 | 6 (31.6%) |
| **Gender, n (%)** |  |
| Male | 16(84.2%) |
| Female | 3 (15.8%) |
| **Tumor size, n (%)** |  |
| ≦5cml | 7(36.8%) |
| >5cm | 12(63.2%) |
| **Differentiation, n (%)** |  |
| well | 5(26.3%) |
| Moderate | 9(47.4%) |
| Poor | 5 (26.3%) |
| **Lymph node metastasis, n (%)** |  |
| No | 18 (94.7%) |
| Yes | 1 (5.3%) |
| **TNM stage, n (%)** |  |
| I-II | 17(89.5%) |
| III-IV | 2 (10.5%) |
