# Supplementary figures and images for: MFSD12 promotes proliferation, metastasis and invasion of hepatocellular carcinoma cells and its potential correlation with HAVCR2/LGALS9 immune checkpoint axis
Source: Front Immunol. 2025 Oct 21;16:1681887. doi: 10.3389/fimmu.2025.1681887 (PMC12582970; doi:10.3389/fimmu.2025.1681887)

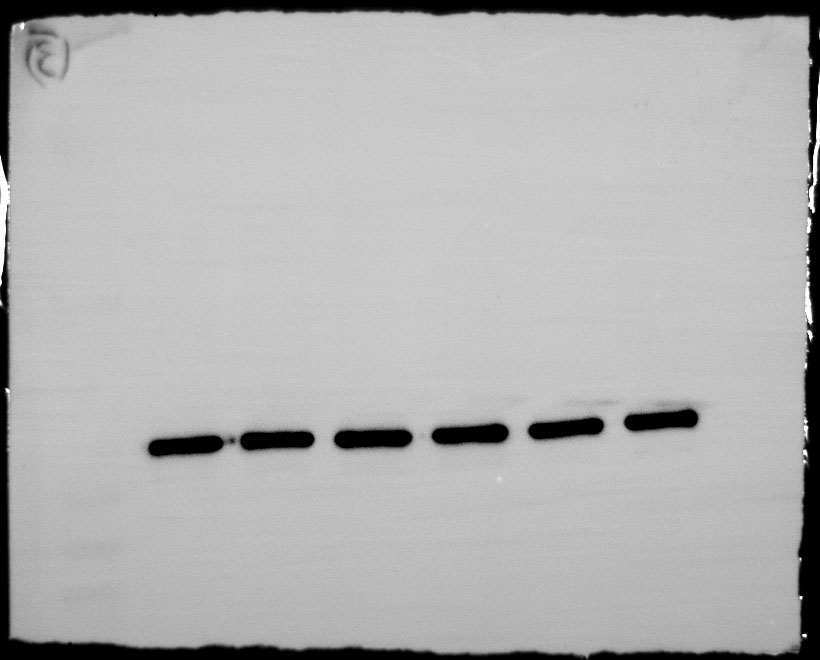

Supplement: Supplementary file 9 [file DataSheet1.zip › Fig12C-GAPDH.jpg]

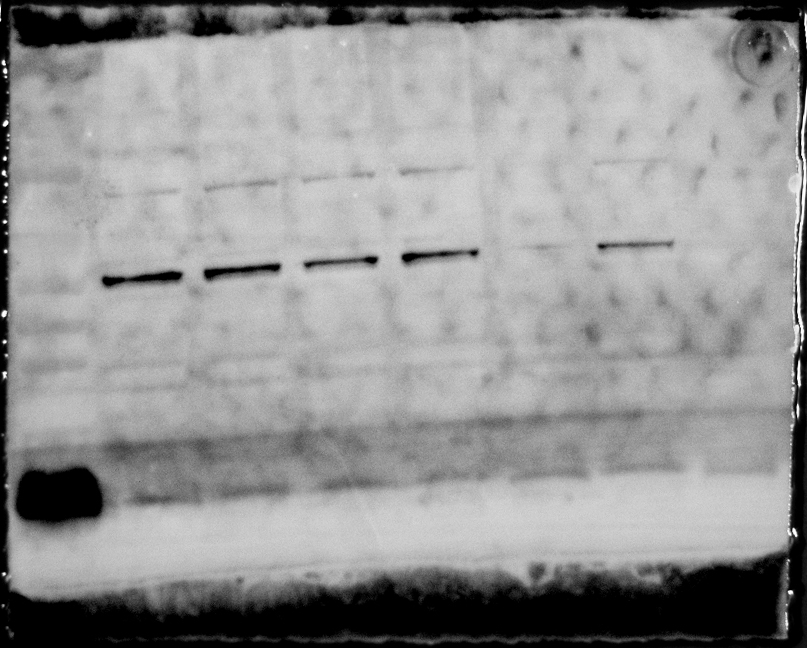

Supplement: Supplementary file 9 [file DataSheet1.zip › Fig12C-MFSD12.jpg]

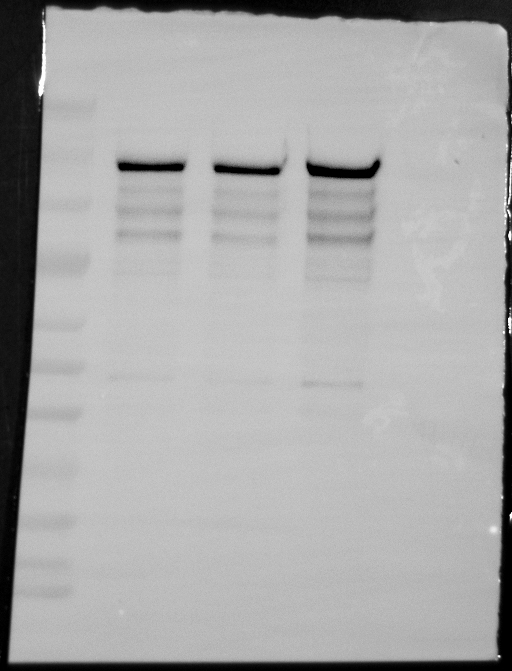

Supplement: Supplementary file 9 [file DataSheet1.zip › Fig12E-E-cad.jpg]

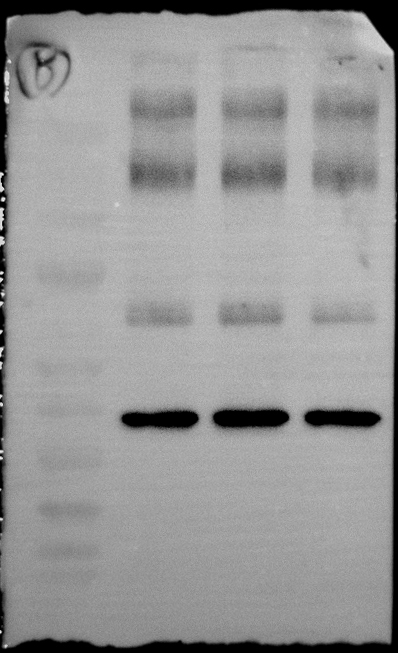

Supplement: Supplementary file 9 [file DataSheet1.zip › Fig12E-GAPDH.jpg]

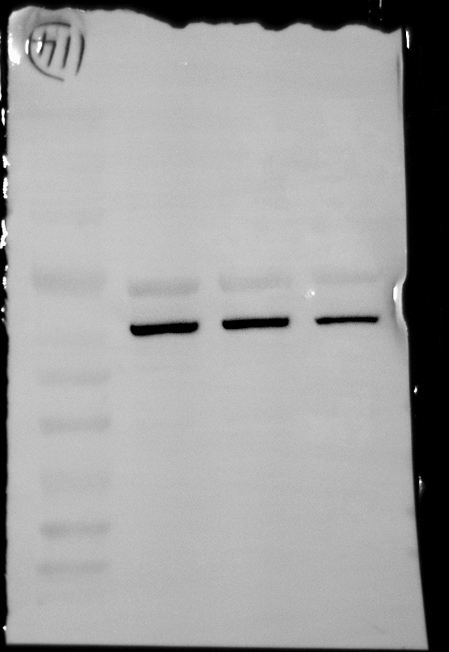

Supplement: Supplementary file 9 [file DataSheet1.zip › Fig12E-HAVCR2.jpg]

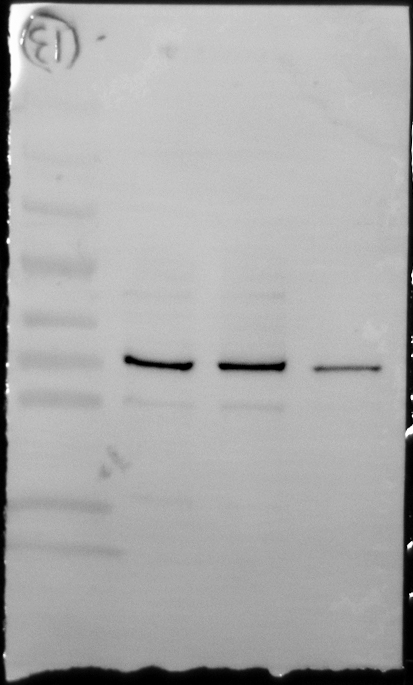

Supplement: Supplementary file 9 [file DataSheet1.zip › Fig12E-LGALS9.jpg]

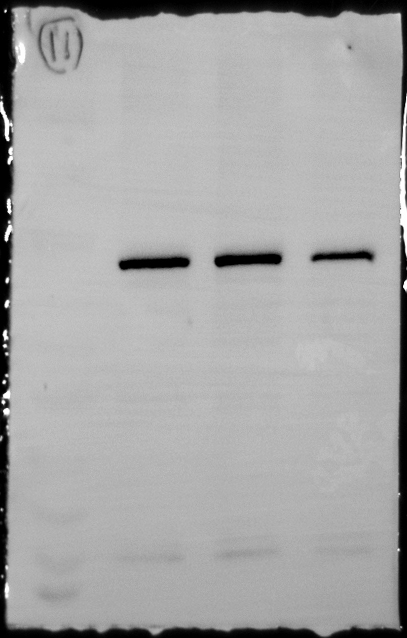

Supplement: Supplementary file 9 [file DataSheet1.zip › Fig12E-MMP-2.jpg]

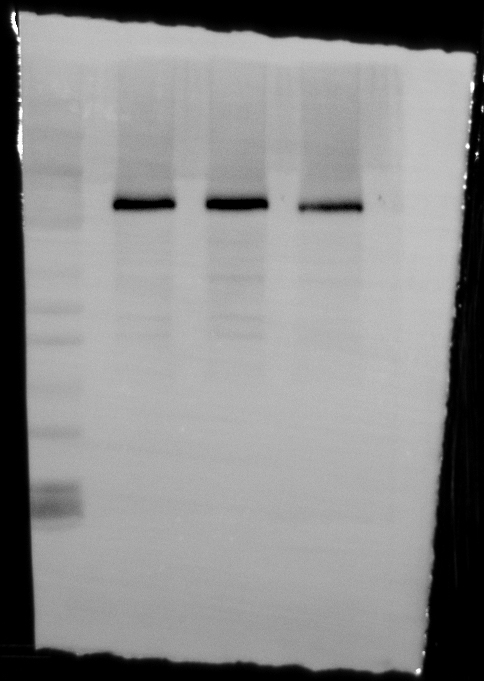

Supplement: Supplementary file 9 [file DataSheet1.zip › Fig12E-MMP-9.jpg]

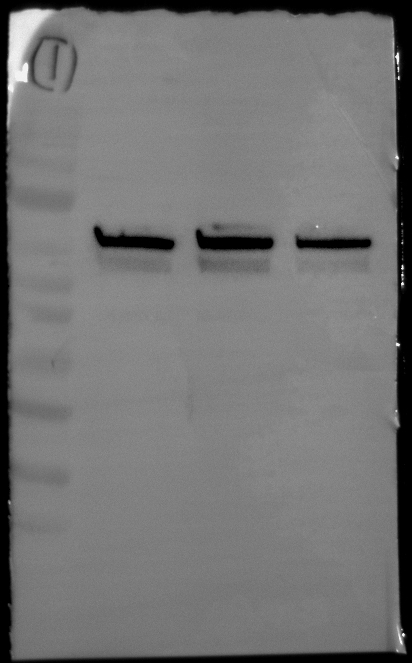

Supplement: Supplementary file 9 [file DataSheet1.zip › Fig12E-vim.jpg]
